# Structure-Dynamics Relationships in Bursting Neuronal Networks Revealed Using a Prediction Framework

**DOI:** 10.1371/journal.pone.0069373

**Published:** 2013-07-25

**Authors:** Tuomo Mäki-Marttunen, Jugoslava Aćimović, Keijo Ruohonen, Marja-Leena Linne

**Affiliations:** 1 Department of Signal Processing, Tampere University of Technology, Tampere, Finland; 2 Department of Mathematics, Tampere University of Technology, Tampere, Finland; Georgia State University, United States of America

## Abstract

The question of how the structure of a neuronal network affects its functionality has gained a lot of attention in neuroscience. However, the vast majority of the studies on structure-dynamics relationships consider few types of network structures and assess limited numbers of structural measures. In this *in silico* study, we employ a wide diversity of network topologies and search among many possibilities the aspects of structure that have the greatest effect on the network excitability. The network activity is simulated using two point-neuron models, where the neurons are activated by noisy fluctuation of the membrane potential and their connections are described by chemical synapse models, and statistics on the number and quality of the emergent network bursts are collected for each network type. We apply a prediction framework to the obtained data in order to find out the most relevant aspects of network structure. In this framework, predictors that use different sets of graph-theoretic measures are trained to estimate the activity properties, such as burst count or burst length, of the networks. The performances of these predictors are compared with each other. We show that the best performance in prediction of activity properties for networks with sharp in-degree distribution is obtained when the prediction is based on clustering coefficient. By contrast, for networks with broad in-degree distribution, the maximum eigenvalue of the connectivity graph gives the most accurate prediction. The results shown for small (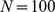) networks hold with few exceptions when different neuron models, different choices of neuron population and different average degrees are applied. We confirm our conclusions using larger (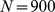) networks as well. Our findings reveal the relevance of different aspects of network structure from the viewpoint of network excitability, and our integrative method could serve as a general framework for structure-dynamics studies in biosciences.

## Introduction

There is a great interest towards understanding the structure of neuronal networks, and ultimately, the full connectome [1], [2]. The network structure lays a foundation to all collective activity observed in the system, and understanding this relationship is relevant both *in vivo* and *in vitro*. Promising experimental attempts have been made in controlling the growth of neurons to produce a pre-designed network structure [3], [4]. If successful, such experiments would inform us on how the collective dynamics of the neurons is influenced by their patterns of synaptic connectivity. However, such information is extremely challenging to obtain using the state-of-the-art equipment due to the complexity of processes involved in neuronal growth. Furthermore, the connectivity patterns obtained using experimental setups are always subject to physical constraints posed by the growing platform of the neurons. For all this, most of the nowadays studies on structure-function relationship in neuronal networks are likely to be conducted *in silico*, where the connectivity can easily be modified and the effect on the network dynamics instantaneously screened.

In the past few decades a lot of theoretical and computational studies on the function of neuronal networks have been carried out in order to examine the behavior of the network under various circumstances and various stimuli. However, in most studies the structure of the network is at least in part based on purely *random networks*, i.e., the far and widely studied Erdös-Rényi networks. These networks are statistically described by a single parameter, namely, the connection probability 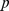, and by far lack any spatial organization. Several studies have revealed the contribution of connection probability to various aspects of neuronal network dynamics, e.g., emergence of large-scale network synchronization [5], [6] the amplitude of fast network oscillations [7], and emergence of spontaneous network-wide bursts [8].

Despite their vast usage, the random networks have been found an insufficient model for the synaptic connectivity in the brain [9]–[12]. Recently, steps toward deeper understanding of the details of the structure and their effects on the dynamics have been made, which is shown by the devotion of a recent special issue in Frontiers in Computational Neuroscience particularly to this topic [13]. The framework of small-world networks [14] which allows varying the proportion 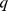 of long-range connections in addition to the connection probability 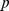 has hitherto been the most studied alternative to Erdös-Rényi networks in models of neuronal networks. Analyses on the effects of the long-range connections on, e.g., oscillation coherency [15], modes of synchrony in models of epilepsy [16], [17], and self-sustained activity [18] have been carried out. However, a range of other extensions to random networks exists as well. The scale-free [19] networks possess a structure that is hierarchical over different scales, and are characterized by power-law distributed degrees. These networks have been applied in a range of neuronal modeling studies due to their resemblance to the hierarchical connectivity of the brain [20]. Nevertheless, the preferential attachment algorithm in [19] (and in most generalizations for directed graphs, e.g. [21]) for generating scale-free topology only uses the first order connectivity statistics, i.e., the number of contacts of the nodes, as the criteria for creating a link. In [22] the effect of second-order connectivity statistics, which can roughly be captured by the widths and correlation of the degree distributions, were studied. Similarly, [23] studied the effect of degree distribution widths through a framework where both degree distributions can be arbitrarily predefined, and the networks are created through random couplings. Both [22] and [23] agree on the significance of the in-degree over the out-degree in influencing the mode of synchrony in the network.

A frequent trend in structure-dynamics studies is to overlook the coeffect of structural measures. The changes in activity are monitored with respect to one graph measure, ignoring the possible mutual changes in other structural measures [24]. In this work we approach this problem by measuring a set of graph properties simultaneously. In addition, we apply multiple network generation algorithms in order to avoid too great correlation between some particular graph measures. As an example, studying only such networks that are described in [14] would bring about a large correlation between geodesic path length and clustering coefficient, which would make it difficult to tell which properties of dynamics are due to the high path length and which are due to the clustering.

The focus of this work is on excitability of spontaneously bursting networks, i.e., on how frequently network bursts occur and of what magnitude they are. Note that we adopt the term *burst* from literature on neuronal networks cultured on a micro-electrode array, where the term is widely used for a short period of high spiking activity (alternative names are many, e.g., *network spike*, *population spike*, and *synchronized spike*) [25], [26]. By contrast, when we refer to a burst of a single neuron, we use the term *intrinsic burst* or *single-cell burst* to make a clear distinction. We apply two point-neuron models, one of which is based on the integrate-and-fire formalism and the other on the Hodgkin-Huxley formalism. In both models, the neurons are connected by chemical synapses expressing short-term plasticity. The synaptic currents (or conductances in the Hodgkin-Huxley type of model) are instantaneous and decay exponentially after a presynaptic action potential. In the case of strong enough recurrent excitation, both models produce network bursts. Our focus is on the regime of spontaneous bursting activity, where the bursting frequency lies between 0 and 60 bursts/min. This is a typical range of bursting in, e.g., cortical cultures [25].

In the present study, we apply a prediction framework to determine the importance of different graph-theoretic measures. Simulations of network activity are run on a large set of different network structures, and measures of both structure and activity are calculated. For each measure of structure we estimate its capability to predict the outcome of the activity properties, and to an extent, its capability to copredict the activity when used together with the other graph measures. We show that the prediction of activity properties in networks with sharp in-degree distribution (binomial) is best when clustering coefficient is used, whereas in networks with broad in-degree distribution (power-law) the predictions based on maximum eigenvalue of the connectivity matrix are the most accurate. Our results could serve as a general guideline for designing experiments in which several but not all aspects of structure are measured. With novel experimental techniques and tools for data analysis [12], [27], graph-theoretic measures of the local connectivity could be estimated without unraveling the whole connectivity matrix, and our results may help to choose those measured aspects.

## Materials and Methods

We restrict our study on networks in which the structure can be fully represented by a directed unweighted graph. We use the notation 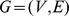, where 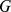 is the graph, 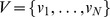 is the set of nodes, and 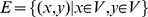 is the set of egdes between the nodes. The connectivity matrix 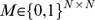 of a graph 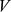 is a binary matrix, where each element 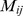 denotes the existence (1) or nonexistence (0) of an edge *from* node 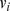 *to* node 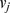. Self-connections are excluded in this work. We call *neighbors* such pair of nodes, that have at least a unidirected edge between them. When no risk of confusion, we use the terms “node 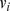
” and “node 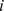
” interchangeably.

### Network structure

We assess network structure using the following graph-theoretic measures.

- *Clustering coefficient* (CC). The local clustering coefficient CC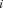 of a node 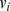 describes the density of local connections in the neighborhood of node 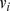. We say that the nodes 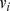, 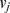 and 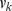 form a triangle if there is at least a unidirected edge between 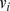 and 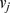, between 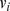 and 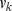, and between 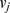 and 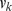. The local clustering coefficient of node 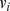 is the number of triangles that include the node divided by the maximum number of such triangles if all neighbors of the node were connected [14], [28]. The directions of the edges are respected, hence changing a unidirected edge to a bidirectional edge doubles the counted triangles that include the considered edge. In mathematical terms, we can write

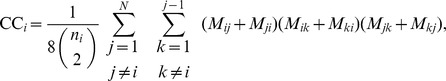
where 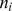 is the number of neighbors of node 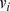. The clustering coefficient CC of the whole network is calculated as the average over the local clustering coefficients of the nodes – only those nodes are taken into account that have more than one neighbor.

- *Harmonic path length* (PL). A geodesic path from a node to another means the shortest traversable path between the two nodes. To calculate the harmonic path length, the geodesic path length PL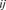 between each pair of nodes 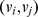, 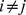 is first calculated, where 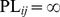 represents the case where no path exists from 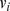 to 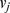. The harmonic path length of the network represents the average distance between two nodes of the network, and is computed as the harmonic mean of the geodesic path lengths [28], [29]:

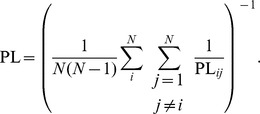

**Figure 1:**
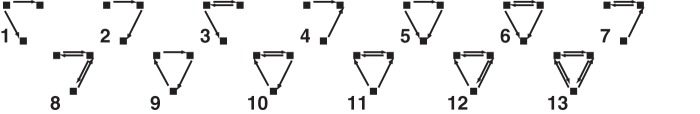
The 13 network motifs of three connected nodes. See [31] for reference.

- *Node-betweenness* (NB). The local node-betweenness 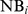 is a measure of centrality of the node 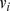. It is calculated as the number of shortest paths that the considered node lies on [28]. If the node lies on a number 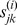 out of 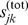 equally long geodesic paths between nodes 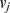 and 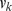, then the increment of this pair of nodes is the fraction of the two quantities. Thus, we can write 
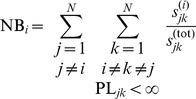
. The node-betweenness NB of the network is the average of the local betweennesses NB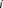.
- *Out-degree deviation* (OD). The sample standard deviation of the realized out-degrees of the nodes.
- *Degree correlation* (DC). The sample correlation coefficient between the realized in- and out-degrees of the nodes.
- *Length-to-self* (LtS). The mean geodesic length to self 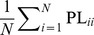.
- *Maximum eigenvalue* (MEig). The largest eigenvalue of the connectivity matrix 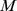. This is always real-valued as the connectivity matrix is non-negative [30].
- *Motif* count (Mot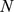, 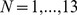). The (absolute) number of different connectivity patterns of triples of nodes [31] (see Fig. 1).

Ideally, to study how measures of structure are linked to measures of dynamics, one would have a direct (possibly stochastic) function from the measures of structure to measures of dynamics. However, to obtain the measures of dynamics or their distributions, a network activity model has to be applied using a certain connectivity graph. Hence, this sort of mapping is not possible unless the measures of structure uniquely determine the underlying graph. To go around this problem, we generate networks with very different structural properties and simulate the neuronal activity in them. We concentrate on a few carefully selected random graph classes that we consider to span wide enough diversity of network types relevant in neuroscience: Watts-Strogatz-type networks (WS), networks with high local feed-forward structure (FF), and networks with high number of loops of certain length (L2,L3,L4,L6).

Let us motivate the choice of these classes. WS networks were first introduced in [14] as a class of networks expressing the small-world phenomenon, and have been extensively used ever since. In neuroscientific studies the WS networks between ordered and random topologies have been proposed as a model for, e.g., optimal signal propagation [15], maximal dynamical complexity [32], and optimal pattern restoration [33]. As for the FF networks, the feed-forward loop is a triple of nodes, 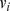, 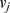 and 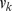, where there is a direct connection from 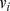 to 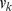, and a “secured” disynaptic connection from 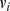 through 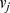 to 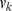. The feed-forward loops have been found more abundant in *C. Elegans* neuronal network than in random networks [31], and their contribution to neural processing has been much studied [34], [35]. We include these networks in the present study as an alternative to WS networks that should show a great number of feed-forward loops and yet lack the spatial structure typical to WS networks. Finally, the loopy networks (L2, L3, L4 and L6) represent a network structure, where the connections are organized such that the feed-back loops of certain length and direction are promoted. The synaptic feed-back projections in general have been suggested as a mechanism for working memory [36], [37]. Several papers discuss the existence of directed loops in the brain: [38] and [39] show that such loops could be produced by rules of spike-timing-dependent plasticity (STDP) in order to promote stability in the network, contradicting with the no-strong-loops hypothesis [40]. The reason to include loopy networks in this study is to address the question whether and to what extent such loops contribute to the dynamics in recurrent neuronal networks.

One of the statistically most dominant properties of recurrent neuronal networks is the connection probability of the neurons. Increasing or decreasing the connection probability has usually major effects on the neuronal activity, which has been discussed in several computational studies, including [7], [41] and [8]. In addition to this, not only the average number but also the variance in number of inputs to the neurons plays a significant role in the synchronization properties of the network [22], [23]. Regarding these facts, we keep the in-degree distributions strictly constrained while studying the other aspects in the network structure. To do this we propose to use the following random graph algorithms in which the in-degree distribution 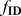 can be explicitly set.

#### Watts-Strogatz [14] algorithm for bidirectional graphs

Initially, the nodes are placed in a metric space of choice. The number of inputs is drawn from 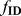 for each node, and that number of spatially nearest nodes are chosen as inputs. Finally, all existing edges are rewired with probability 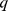 such that the postsynaptic node is held fixed but the presynaptic node is picked by random. We call these networks WS1 and WS2 networks, where the number 1 or 2 tells the dimensionality of the manifold where the nodes lie. In WS1 networks the nodes are placed on the perimeter of a ring, while in WS2 networks the nodes are placed on the surface of a torus. To be more specific, in the ring topology the nodes are placed into a ring in 2D plane as 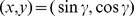, where 
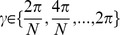
. Similarly, in the torus topology the 2D grid is nested into 4D space as 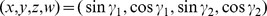, where 
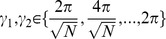
, given that 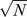 is an integer. In both topologies the Euclidean distance is used as the metric. We refer to the limit topologies of Watts-Strogatz networks with zero rewiring (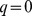) as *locally connected networks* (LCN1 and LCN2).

#### Scheme for generating graphs with high local feed-forward occurrence

For each node the number of inputs is drawn from 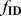. The inputs are selected sequentially for each node. For the first node, the inputs are selected by random. For the next ones, the inputs are selected in such a way, that the emergence of feed-forward motifs is pronounced. This is done by giving higher weights to the nodes that project disynaptically to the considered node than to the others. A detailed scheme for generating these networks is given in Algorithm S1 in File S1. We refer to these networks by acronym FF. Note that this is not to be mistaken for the general term *feed-forward networks* in the meaning of opposite for recurrent networks. In this work all considered networks are recurrent.

#### Scheme for generating graphs with high occurrence of loops of length 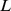


For each node the number of inputs is drawn from 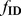. The edges are set one by one until each node has all its inputs selected. In the selection of presynaptic nodes, the emergence of loops of length 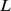 is promoted, while the addition of edges that shorten these loops is discredited. This is done by giving different weights to the nodes depending on the shortest path from the considered node to the candidate nodes. See Algorithm S2 in File S1 for the detailed algorithm. The resulting networks are rich in recurrent synfire chains of length 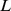. This is however conditional to the choice of the in-degree distribution: If the number of connections is too great, the excessive edges have to create “shortcuts” into the loops. In this work we refer to these networks with acronym L2, L3, L4 or L6, depending on the promoted length of loops.

MATLAB functions to generate these networks are given in ModelDB entry 147117. Each of these algorithms can be used to generate both networks where the definitive property of the respective network is very pronounced, networks where the strength of that property is zero (random networks), and networks that lie in between these extremes on a continuous scale. We denote this *strength parameter* by 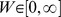. In Watts-Strogatz networks, we draw the relation between the rewiring probability 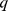 and the strength parameter as 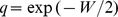. Hence, in all network classes 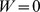 produces strictly random networks (RN) and 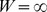 produces the other extreme of networks.

In addition to these networks, we consider biologically realistic 2-dimensional neuronal networks. To generate these, we use the NETMORPH simulator [42] with the model parameters taken from [43]. NETMORPH simulates the growth of dendrites and axons in a population of neurons and outputs the sites of potential synapses. The potential synapses are formed when an axon and a dendrite of distinct neurons come close enough to each other. To remove the effect of boundaries, we place the somas randomly inside a square-shaped box, and the neurites that grow outside the box are considered to appear on the opposite side of the box. For each simulation, we form the connectivity graph from the simulation result once the required amount of connections has been reached. We omit the question of to which degree the potential synapses become functional synapses and consider every potential synapse as an edge. Multiple synapses with the same pre- and postsynaptic neurons are considered as one edge. The in-degree distribution of these NETMORPH networks cannot be explicitly set, but it is fairly well approximated by binomial distributions (see Fig. S1 in supporting information). In the forthcoming sections, we abbreviate the networks obtained with the NETMORPH simulators as NM.

**Figure 2:**
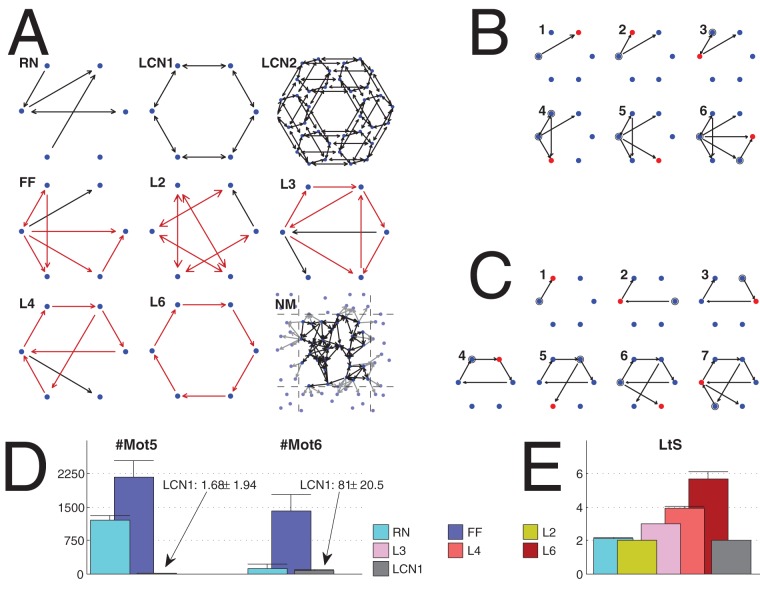
Illustration of network classes. **A:** Examples of the extreme network types used in the present work. Network size 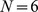, except in LCN2 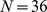, and in NM 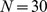. The red arrows highlight the definitive properties of the networks. In NM the connections whose post-synaptic node lies across the box boundaries are replaced by a link to a copy of the post-synaptic node (plotted with gray at a corresponding location outside the box). **B, C:** Illustration of the generation of FF (B) and L4 (C) networks. The red dots show the node that has recently been added the inputs, and these inputs are in turn highlighted by circles. The number at the upper-left corner of each graph shows the iteration number. **D**: Mean and standard deviation of the number of motifs 5 (left) and 6 (right) in different extreme network types (RN, FF, and LCN1). The FF networks possess the greatest number of both these two motifs. The low number of these motfis in LCN1 networks is explained by the fact that they contain much more highly connected motifs (motifs 12 and 13) due to their locally coupled design. **E**: Mean and standard deviation of the length-to-self measure in different extreme network types (RN, L2, L3, L4, L6, and LCN1). The loopy networks L2, L3, L4, and L6, express a value of LtS near to the corresponding length of the promoted loop. In both D and E, all networks are of size 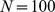 and their in-degree distribution is binomial with 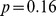. Statistics are computed from 150 independent samples.

The different network classes are illustrated in Fig. 2. In addition, iterations for the generation of extreme FF and L4 networks are shown. Furthermore, a set of graph measures in extreme FF, L2, L3, L4 and L6 networks are shown. These statistics, compared to the corresponding statistics in random and locally connected networks, reveal that the algorithms indeed produce networks with the desired properties. Further properties of the networks are shown in Figs. S2, S3 and S4, and discussed in Section S2 in File S1.

### Neuronal dynamics

We apply two neuron models with rather different intrinsic dynamics. The first one is a leaky integrate-and-fire model with short-term plasticity [44], and the second one is a Hodgkin-Huxley type of model with four ionic and three synaptic currents [45]. In the latter we import a model of synaptic short-term plasticity from [46]. In both models we input a stochastic white noise term into the membrane potential of the neurons to make them spontaneously active. The models are described in detail in Section S1.3 in File S1.

We refer to the first model as *LIF* model and to the latter as *HH* model throughout this work, although they are extensions of the ordinary leaky integrate-and-fire and Hodgkin-Huxley models. These two models were chosen to represent both a simple model that can easily be extended to larger networks, and a more biophysically detailed model that can be extended to study the effect of, e.g., various neurotransmitters and modulators on network activity. The latter was introduced as a model for studying synchronization in low extracellular magnesium concentration, but it allows the use of higher concentrations as well. Here, we use a value 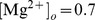
mM, which is in the range of magnesium concentrations normally used in studies of neuronal cultures (see, e.g., [25]).

Network bursts could be produced with simpler models that do not consider short-term plasticity, e.g., by using widely applied models of balanced excitation/inhibition [47] or Markov binary neurons [48]. The ending of the bursts in these models is dependent on the activation of the inhibitory population, which returns the elevated firing activity to a baseline level. By contrast, applying short-term depression to the excitatory synaptic currents allows the emergence of network bursts in both excitatory-only (E) and excitatory-inhibitory (EI) networks [44]. This is favorable, as the experiments carried out on neuronal cultures show that network bursts cease even in the pathological case of blocked inhibition (see, e.g., [49] and [26] for spinal cord cultures and [50] and [51] for cortical cultures). In this work, we study the bursting dynamics of both E and EI networks, and hence, we employ the short-term depressing synapses in both cases. In the EI networks, the structure is first generated using one of the network generation schemes and then 20% of the neurons, randomly picked, are assigned as the inhibitory population. The network size is 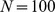 unless otherwise stated.

As a major simplification to reality, we consider the synaptic transmission to be instantaneous. The transmission delays and their effect on neuronal network dynamics have been under wide examination (see e.g. [52]) and have been shown to play an important role in various contexts. Their inclusion can be, however, carried out in multiple ways. For instance, in WS1, WS2, and NM networks the long-range connections should have longer delay parameters than the local connections (see e.g. [53]), whereas for other network types such distance-delay relationship cannot be straightforwardly defined, and hence, different approaches should be tested. In this work we restrict our study to non-delayed networks in order to avoid excessive simulations.

The networks are set into a regime of spontaneous network bursting. This is done by tuning the synaptic weight 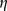 (see Section S1.3 of File S1) so that the moderately connected networks (RN, 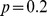, binomial in-degree) show a bursting frequency of 10 bursts/min. These values are in the range of connectivity and bursting activity in a typical cortical culture [25]. For the applied proportions of excitatory and inhibitory neurons and model parameters, we found that the mean bursting frequency is a monotonically increasing function of the synaptic weight in the regime of interest (0–60 bursts/min), and hence we use the bisection method to find the proper synaptic weight.

**Figure 3:**
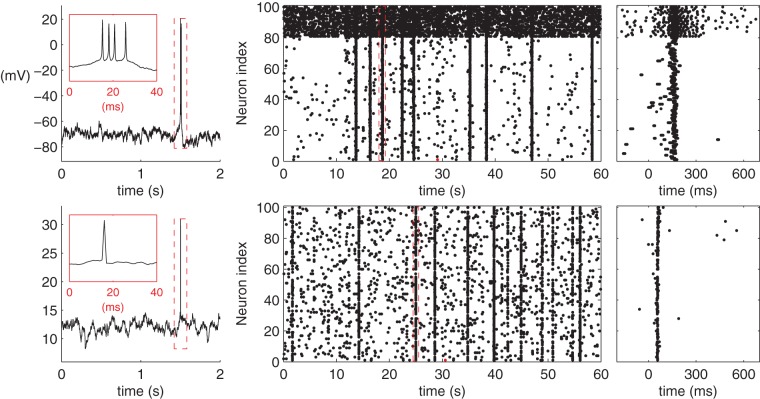
Illustration of the HH (upper panels) and the LIF (lower panels) model dynamics. **Left:** Single cell membrane potential with the spike magnified in the inset. The membrane potential at the time of spike in the LIF model explicitly set 30 mV for the sake of illustration. **Middle:** Network spike train in an excitatory-inhibitory RN with 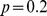 connectivity and binomial in-degree distribution. The upmost 20 neurons represent the inhibitory population. The red spike corresponds to the (first) spike shown in the left panel, and the burst with the red borders corresponds to the burst shown in the right panel. **Right:** The selected burst highlighted.

For each network simulation the spiking activity is solved for a one minute period (in fact for 61 s, but the first second is neglected for a possible transition stage). The model parameters and initial conditions for both models are described in Section S1.3 of File S1. The code files to carry out the simulations in PyNEST [54] (LIF model) and MATLAB (HH model) are given in ModelDB entry 147117. Fig. 3 illustrates the typical dynamics for a single neuron and a network of neurons.

**Figure 4:**
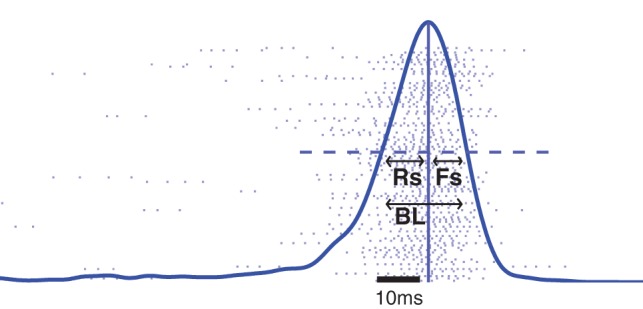
Illustration of the burst profile attributes. The shaded dots represent the spikes of the excitatory neurons. The thick blue curve represents burst profile, i.e., the smoothened firing rate curve. The time instants when the burst profile for the first and the last time crosses the value of half of the maximal value (shown with horizontal dashed line) are identified. The distances of these time instants from the time instant of the maximal firing rate (vertical line) are the lengths of the rising (Rs) and falling (Fs) slope. The burst length (BL) is the sum of these two attributes. The network activity in this figure is simulated with the HH model, and the structure of the underlying network is a RN with binomial in-degree distribution, 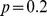. Scale bar (black) 10 ms.

Activity in a bursting network can be characterized by the quantity and quality of the network bursts. We employ the burst detection scheme applied in, e.g., [55] and [56]. The spikes are first divided into separate network bursts using a maximal inter-spike interval of 25 ms. This means that two consecutive spikes belong to the same network burst if and only if their distance is 25 ms or less. Those bursts which consist of less than 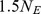 (with HH model) or 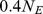 (with LIF model) spikes, where 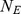 denotes the size of the excitatory population, or in which less than 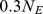 individual neurons contributed to the burst, are disregarded. Further, a *burst profile* is created by convoluting the population spike train in the range from the first to the last spike of the burst with a Gaussian with deviation 2.5 ms. The length of the *rising slope* and the *falling slope*, i.e., the halfwidths of the burst profile, are calculated with a resolution of 0.25 ms. These measures are illustrated in Fig. 4. We consider the summed value of these two measures the *length of the burst*. This measure is more robust to addition of a single spike to the burst than the absolute duration of the burst, which is calculated as the time from the first spike to the last spike of the burst. To further characterize the burst, we consider the number of spikes in a burst, which we refer to as the *burst size*. To average the network activity over a one minute simulation, we use the median burst length and median burst size. An important characteristic of the network activity is also the *burst count*, i.e. the number of bursts during the time of simulation, which has been shown to vary substantially in spontaneously active networks with different structures [57]. In addition, we consider the total *spike count* of the network during the one minute simulation as an indicator of the overall amount of activity.

All above activity measures are calculated from the population spike train of the network. In the LIF model the spike trains are given explicitly by the model, but in the HH model they have to be extracted from the time series of the membrane potential. In this work, we consider any *local maximum* of the membrane potential above the threshold of −30 mV a spike. It should be noted that due to the Brownian noise injected to the membrane potential, we only consider local maxima at the resolution of 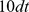, where 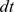 is the simulation time step. This means that the time instant 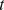 is considered a local maximum if and only if 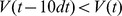 and 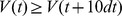. Given the simulation time step 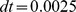
ms, this resolution was found scarce enough to prevent the noisy fluctuation of the membrane potential from being registered as spikes but on the other hand fine enough to correctly detect spikes in an intrinsic (single-cell) burst. The chosen threshold potential, -30 mV, is robust. In a RN with binomial in-degree distribution (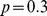), the change of 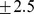
mV in the threshold potential had no effect on the detected spikes, and a change of 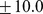
mV changed the total number of detected spikes by less than 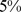.

### Structure-dynamics analysis

Using the above methods, a realization of activity properties can be obtained for any given connectivity graph by simulating one of the two neuron models and performing the burst detection. In purely excitatory networks the graph properties are extracted using the entire network, while in EI networks only the excitatory-excitatory part is considered. The activity properties are likewise calculated from the excitatory population merely.

Throughout this work, we divide the data into 24 *simulation settings*, as listed in Table 1. The networks in each simulation setting have a fixed average connection probability 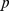 (0.16, 0.2, or 0.3), a fixed shape of in-degree distribution (BIN as binomial or POW as power-law), a fixed choice of population (E or EI) and a fixed choice of model of dynamics (HH or LIF). Hence, all variation in activity properties between networks that belong to the same simulation setting is an effect of the network structure only. For each setting we generate a series of network structure realizations and for each of these we simulate a one minute sample of activity. The chosen network types are FF, WS1 and WS2 networks with 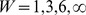, and L2, L3, L4 and L6 networks with 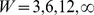. In addition, RNs are included, and NM networks are considered in settings with binomial in-degree distribution, which makes the total number of essentially different types of network structure 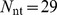 (power-law) or 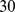 (binomial).

**Table 1:** The list of the 24 simulation settings.

HH												LIF											
E						EI						E						EI					
BIN			POW			BIN			POW			BIN			POW			BIN			POW		
0.16	0.2	0.3	0.16	0.2	0.3	0.16	0.2	0.3	0.16	0.2	0.3	0.16	0.2	0.3	0.16	0.2	0.3	0.16	0.2	0.3	0.16	0.2	0.3

The first row denotes the model of dynamics, and the second row shows the choices of population. For each combination of these one may freely choose the shape (third row) and connection probability (fourth row) of the in-degree distribution.

We use two methods for the data analysis, namely, a correlation analysis and a prediction framework. We use the first to restrict the number of analyses to be done with the latter. The correlation coefficient between activity property and graph property is calculated for each simulation setting separately as
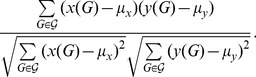
(1)In this notation, 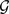 is the set of networks (we use terms network and network realization interchangeably here) belonging to a said simulation setting. The term 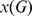 is a graph property of network 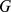, while the term 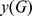 is an activity property obtained from a neuronal simulation done on network 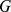, and 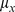 and 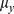 are the corresponding average values.

The correlation analysis is useful as a first approximation of the relationship between the graph measures and activity measures, but it only sheds light on the linear pair-wise dependence between the measures. We apply a prediction framework to answer the question: Which graph measures are the most important when aiming to predict the activity in the network? To do this, we divide the data into a teaching data set and a target data set. The teaching data set consists of 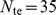 networks for each of the 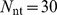 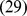 network types, while the target data set contains only 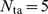 repetitions. An affine predictor
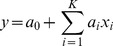
(2)is built using the considered activity properties 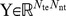 and the 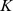 chosen structural properties 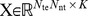 that are extracted from the teaching data. We include the realized average degree in the structural measures in order to compensate for the variety caused by in-degree variance, and hence, we always have 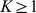. Least mean squares is used to solve the predictor coefficients, i.e.,



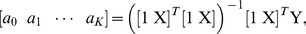
where 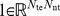 is a vector consisting of 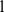
's.

The activity properties of the target data set can be predicted using Eqn. 2 for each of the 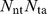 networks, and the prediction error can be calculated as the average absolute difference between the predicted and actual value of the activity property. The prediction is repeated for 10 times in total. During the repetition the target data are regenerated, but the teaching data are resampled from a total pool of 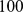 samples of each network type. The error distribution for a given predictor, i.e., a predictor that uses a chosen set of structural measures, is compared to the error distribution of other predictors. This is done using Mann-Whitney's U-test, which tests the null hypothesis that the medians of the distributions are equal.

It should be noted that we do not use the term “predict” in the meaning of forecasting the future based on the past. Instead, the task of the predictor is to estimate the outcome of an activity property in a separate, unknown network when only some aspects of the network structure are known to the predictor. This is closely related to *classification tasks*, but as the outcome of the predictor is a continuous value instead of discrete, it is best described by the term *prediction task* [58].

## Results

**Figure 5:**
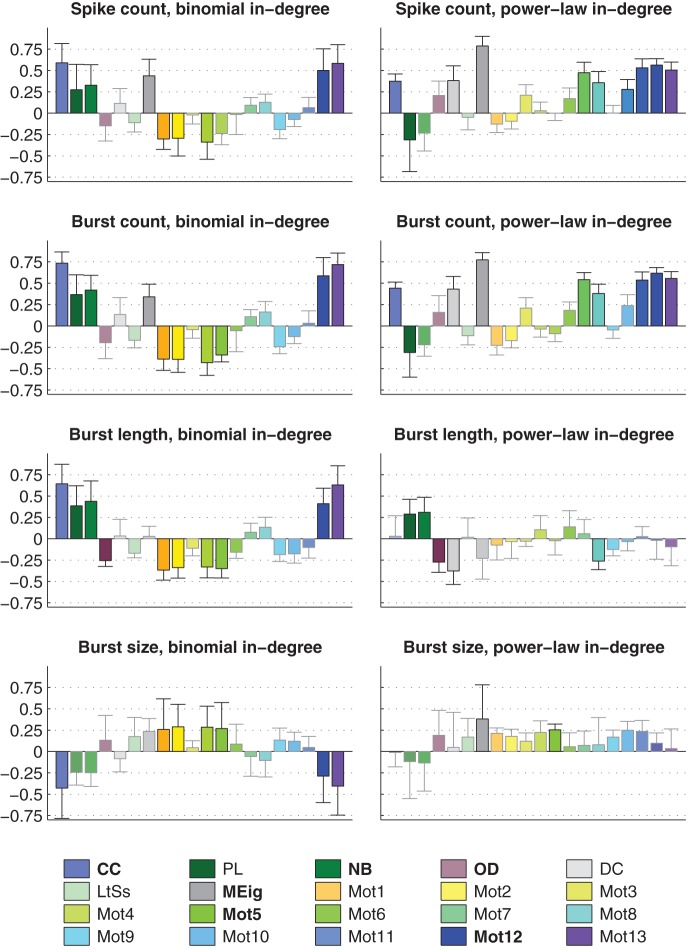
The mean and standard deviation of the correlations between graph measures (see legend) and the activity measures (spike count, burst count, burst length, and burst size). The Eqn. 1 is used for calculating the correlation coefficients for each simulation setting separately. The set of networks 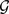 consists of 150 repetitions of each of the 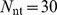 (29) network types. In the panels on the left the mean correlation is taken over correlation coefficients in the twelve simulation settings that use binomial in-degree distribution, while in the panels on the right the twelve simulation settings with power-law distribution are used. The faded bars represent pairs of measures with absolute mean correlations smaller than 0.25. The graph measures that were finally chosen for structure-dynamics study are bolded in the legend.

As a first step for understanding the structure-dynamics relationships in bursting neuronal networks, we estimated the correlations of graph-theoretic measures and activity properties. Fig. 5 shows the correlation coefficients between the considered graph measures and measures of activity. We first calculated the correlation coefficient between all pairs of measures in each simulation setting by Eqn. 1. We then computed the mean and standard deviation of the obtained correlations, taken over the twelve simulation settings with the same shape (binomial or power-law) of in-degree distribution. We focus our analysis on those graph measures that at least *for some activity property* gave an absolute mean correlation *greater than 0.25 in both binomial and power-law settings*. Namely, they were CC, PL, NB, OD, MEig, Mot5, Mot12, and Mot13. However, CC and Mot13 were very strongly correlated with each other (correlation coefficient between these measures ranges from 0.85 to 0.99 in the 24 simulation settings, mean 0.94). This was the case also between PL and NB (0.91 to 0.99, mean 0.95), which is backed by the analytical derivations shown in section S2.4 in File S1. Hence we disregarded PL and Mot13 whenever NB and CC were considered. Other pairs of measures were considerably less correlated: The strongest correlation among the remaining measures was between CC and Mot12, where the correlation coefficient ranged from 0.59 to 0.87, mean 0.77. It should be noted that MEig was to some extent correlated with the average degree of the network (correlation coefficient on range from 0.63 to 0.89, mean 0.79) as predicted by mean-field approximations in [59]. In our framework the mean degrees 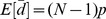, where 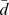 represents the average degree of the nodes, were held fixed between compared networks. However, drawing from the in-degree distribution resulted in some variance in the network structure. In the case of binomial in-degree this variance was negligible (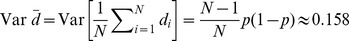 for 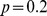), where 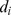 represents the in-degree of a single node, but in networks with power-law distributed in-degree (with 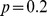) it was empirically found as large as 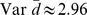. This variance had to be taken into account explicitly in the analyses of the following sections.

Similarly to structural measures, there was redundancy in the activity measures. Naturally, the total spike count was largely dictated by the product of burst count and median burst size: Correlation coefficient between these measures ranged from 0.866 to 0.999 with mean 0.978. In most of the following analyses we disregarded one of these measures, namely the burst size, due to its small coefficient of variation (mean CV 0.16, whereas those of spike count and burst count were 0.46 and 0.62, respectively). The low variance in burst size was also reflected in a high correlation between the spike count and the burst count (correlation coefficient ranged from 0.532 to 0.998, mean 0.918). Between other pairs of activity measures, the correlation coefficient ranged from negative to positive values. Hence, we also neglected the spike count in most of the forthcoming results and considered it to behave to a great degree similarly to the burst count.

### Clustering coefficient regulates the bursting properties in networks with binomial in-degree distribution

**Figure 6:**
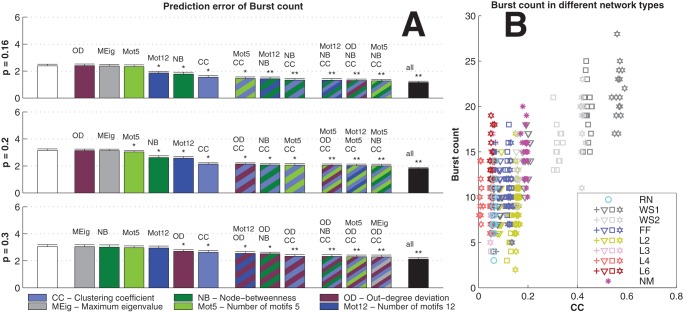
Burst count is best predicted using CC when in-degree is binomial. **A:** The bars in the three panels show the prediction errors (mean + std, 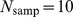) when different graph properties are used as predictors. The errors are calculated as the difference between the predicted and realized number of bursts. The HH model is used in purely excitatory networks with binomial in-degree distribution and average connectivities 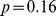 (upper), 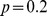 (middle) and 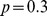 (lower). The leftmost bar (white) shows the mean prediction error of the null predictor. The next group of six bars shows the prediction errors of predictors with an additional graph property, in the order of descending prediction error. The next three bars correspond to the best three predictors that use two graph measures, and the next three bars correspond the ones with three measures. The final bar (black) shows the prediction error of the predictor that uses all available structural data (20 measures in addition to the realized degree). If the error is significantly (U-test, 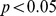) smaller than that of the null predictor, an asterisk (*) is plotted, whereas (**) announces that the error is also significantly smaller than that of the best predictor using one graph property (here always CC). The more graph measures are included in the prediction, the more accurate the prediction is. The error values shown are absolute: For reference, the mean burst counts (averaged over all network types) in the three connection probabilities are 3.4 (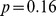), 11.7 (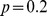) and 31.5 (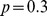). **B:** Values of burst count plotted w.r.t. CC in networks with connection probability 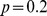. Different network classes are plotted with different colors, and the different markers of WS1, WS2, FF, L2, L3, L4 and L6 networks represent different values of parameter 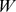 (‘+’ for the lowest value, and stars for the highest value). One finds that the burst count ascends with increasing CC, as suggested by the positive correlation of burst count and CC in Fig. 5.

To further analyze the dependency between activity and graph properties, we applied the prediction framework for different activity properties in different simulation settings. Fig. 6 shows the prediction errors of the burst count in simulation settings with excitatory-only networks, binomial in-degree distribution, and HH model. The error distribution (mean, std) is plotted for different predictors. One finds that predictors using CC are significantly better than the “null” predictors (the predictors where 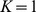, i.e., only the realized degree is used in the prediction). In the dense connectivity simulations (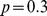) the OD performs approximately equally well, but in other connectivities the effect of OD is insignificant. The distribution of the values of burst count with respect to the values of CC are illustrated for the 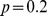 case.

**Figure 7:**
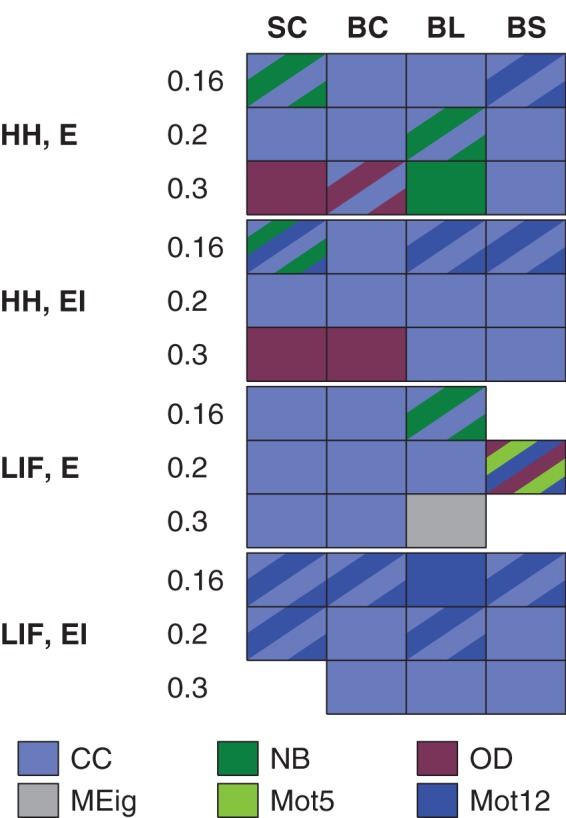
CC gives the best prediction in most simulation settings with binomial in-degree distribution. The best predictor is named for each simulation setting (the 12 rows) and each activity property (the 4 columns: spike count, burst count, burst length, and burst size). The color of the box indicates the graph measure that gives the smallest prediction error when used together with the realized degree of the network. Boxes with stripes mean that another or several other graph measures have statistically indistinguishable error compared to the best predictor. The missing boxes indicate that no predictor is statistically better than the null predictor (U-test, 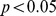). CC wins 38 unique or shared best performances, as the respective numbers for Mot12, OD, NB, Mot5 and MEig are 11, 5, 5, 4, 1 and 1.

**Figure 8:**
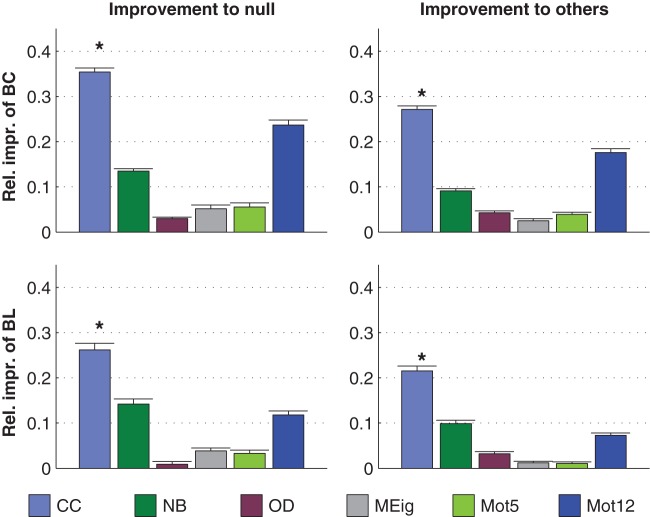
CC brings greatest improvements to the predictions of burst count (BC) and burst length (BL) in networks with binomial in-degree distribution. **Left:** The y-axis shows the relative improvements with respect to null prediction. For each simulation setting, the prediction error for null predictor and the predictor with a considered graph property 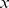 are calculated, using 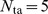 and 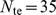. The relative improvements 
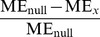
 are averaged over all 12 simulation settings with binomial in-degree distribution. Plotted is the improvement (mean and std) for 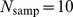 repetitions. The improvement obtained by using CC (*) in the prediction is significantly greater than that obtained by any other single graph measure. **Right:** The y-axis shows relative improvements with respect to prediction by other graph properties. As an example, the first bar shows the relative improvement 
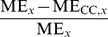
, averaged over graph properties 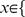
NB,OD,MEig,Mot5,Mot12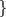. The improvements are further averaged over all 12 simulation settings, and the mean + std of 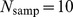 repetitions are shown. The procedure is similar for the other bars. The improvement obtained by using CC (*) in the coprediction is significantly greater than that obtained by any other graph measure (U-test, 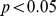).

The dominance of CC in prediction of activity properties can be observed for all simulation settings with binomial in-degree distribution. This is confirmed in Fig. 7, where the best predictor was named for the prediction of each activity property in each of the twelve simulation settings. Furthermore, Fig. 8 shows the averaged improvements that were obtained by using the said graph measures in the prediction of burst count and burst length. One can observe that the predictions were best improved from both the null predictor and from a predictor using an arbitrary other graph measure by including CC in the predicting graph measures. The next best predictors were Mot12 and NB. The improvements obtained by adding OD, MEig and Mot5 were small. The improvement in the prediction was most substantial in the case of burst count: By using only one predicting graph measure (CC) the error was reduced by up to 35% on average, while the corresponding prediction error reduction for burst length was on average 26%. The predictor using all available structural measures reached corresponding percentages of 49% for burst count and 45% for burst length (data not shown).

### Maximum eigenvalue is the best predictor of activity when in-degree is power-law distributed

**Figure 9:**
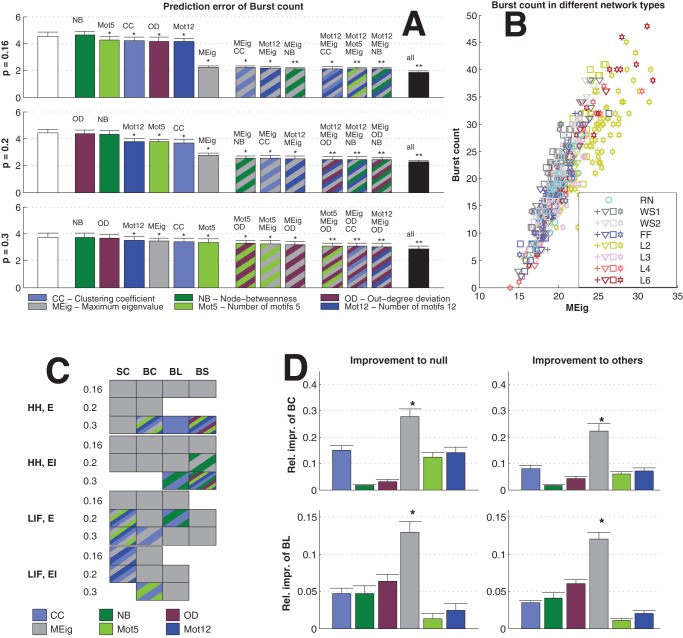
Maximum eigenvalue gives the best prediction of the activity properties across the 12 simulaton settings with power-law distributed in-degree. **A:** Prediction errors of burst counts in simulation settings with the HH model, purely excitatory networks and power-law distributed in-degree. The average burst counts in the three simulation settings are 8.7 (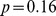), 20.0 (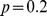) and 40.3 (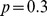). See Fig. 6 for details. **B:** The distribution of the values of burst count in different networks with 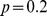, plotted against the MEig of the underlying graph. See Fig. 6 for details. **C:** MEig is the best predictor of activity in most cases (32 unique or shared best performances, in comparison to 12 for CC, 6 for Mot5, 7 for Mot12, 3 for NB, and 2 for OD). See Fig. 7 for details. **D:** In the prediction of burst count and burst length, MEig brings the greatest improvement both to the null predictor and to the predictors based on other graph measures. See Fig. 8 for the details.

We repeated the analyses carried out in the previous section, now using networks with power-law distributed in-degree. The results were substantially different: Changing between excitatory-only and excitatory-inhibitory networks, between different activity models, or even between different connection probabilities did not affect the overall significance of the graph measures in the prediction of activity measures as much as the choice of in-degree distribution did. Fig. 9 shows the statistics corresponding to those shown in Figs. 6, 7 and 8.

One observes a great improvement in prediction by the inclusion of MEig in Fig. 9. This effect was most evident in the networks with the lowest connection probability (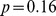, Fig. 9A) where the bursts were most rare (see Fig. S5). Fig. 9C shows the dominance of MEig across activity properties and all simulation settings with power-law distributed in-degree. The prediction errors of burst count and burst length were decreased from null predictions on average by 28% and 13% by the inclusion of MEig (Fig. 9D), respectively. The corresponding percentages for the predictor using all structural data were 41% and 34% (data not shown), which suggests that it be useful to employ more than one structural measure especially in the prediction of burst length.

In these analyses, the realized degree was included in all the predictions in order to cancel the effect of correlation between MEig and the average degree. If the degree was neglected, the effect of MEig was even more pronounced. By contrast, the exclusion of degree from the predictions of activity measures in networks with binomial in-degree had no notable effect due to the low intrinsic variance in the degree. Furthermore, the results stayed the same when a neural network predictor (default feed-forward backpropagation network in MATLAB) was used instead of linear predictor. If a diagonally quadratic predictor (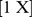 replaced by 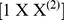 where 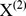 is the element-wise second power) was used, the improvements by the addition of NB and OD were slightly increased, however retaining the statistical dominance of CC and MEig in the prediction of all activity properties (data not shown).

We carried out corresponding simulations with larger networks, 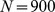. We used the LIF model and excitatory-only networks, and varied the in-degree distribution. Fig. S6 shows the representative data about large network activity and the predictor performances. Our conclusions hold with large networks as well: The activity properties in networks with binomially distributed in-degree can be best predicted with CC, whereas the activity in networks with power-law distributed in-degree can be best predicted using MEig. In addition, we ran longer, 5 minute simulations using the LIF model networks with the normal network size 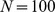 (data not shown). Our results remained qualitatively the same and confirmed that the shorter (1 minute) simulations give statistically significant results in spite of the large variability in the activity properties.

## Discussion

In this work we studied the graph-theoretic properties of several types of networks, and searched for the most relevant aspects of network structure from the viewpoint of bursting properties of the network. Our framework for network generation allows the use of arbitrary in-degree distribution. This allows a fair comparison between the dynamics of different network types, given that the distribution of in-degree plays a crucial role in determining the network dynamics [22]. The relevance of the graph-theoretic properties of the network are assessed in a prediction framework. We calculated how much the prediction of an activity property, such as burst count or average length of a burst, is improved when the prediction is based on a given graph property. We found that in the networks with sharp (binomial) in-degree distribution CC plays the most crucial role (Figs. 7 and 8), whereas in networks with wide (power-law) in-degree distribution MEig is the most relevant graph property (Fig. 9C–D). These results are consistent with few exceptions in the twelve combinations of the two neuron models (HH and LIF), two choices of neuron population (excitatory-only and excitatory-inhibitory), and three connection probabilities (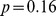, 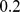, and 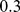). The simulations were run using small (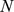 = 100) networks due to the high computational load needed for generation and analysis of a large enough data set, but we confirmed our main findings using a small subset of simulations with larger (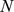 = 900) networks (Fig. S6).

Our framework that combines the use of multiple different types of networks allows the concurrent study of importance of different graph measures, namely CC, PL, NB, OD, DC, LtS, MEig, and Mot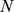, 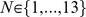. The structural measures were chosen according to the mainstream trends in the theory of complex networks. MEig, which is closely related to DC [59], has been previously shown to play a crucial role in the synchronization of the network [60], [22] using the measure of complete synchrony or its derivatives. We found similar results for networks with power-law distributed in-degree. We considered the measures of activity that we find the most describing of spontaneously bursting networks, namely, the spike count, burst count, burst length, and burst size. The computational framework allows the study of many other aspects of dynamics, e.g., Lyapunov exponents during the onset of the bursts, but in this work we restrict to those measures of activity that can be obtained experimentally as well. The effect of variable average degree of the network that is due to the high variance of the power-law distribution was compensated for by including the realized degree into all predictions. Excluding the degree from the prediction would further stress the importance of MEig in prediction (data not shown). Moreover, the results in Fig. 8 stay qualitatively the same if MEig is replaced by DC (data not shown). Furthermore, the domination of MEig and DC remains even if all 20 graph measures are included in the study or if quadratic or mathematical neural net based prediction is used instead of the affine predictor.

However, MEig (or DC) is not the only structural measure that is determinant of the network activity. The sharp in-degree distribution in networks with binomially distributed in-degree results in little variation of MEig and DC compared to networks with power-law distributed in-degrees. At the same time, the measures of network dynamics, such as spike count and burst count, show comparable – although somewhat smaller – ranges of values for both networks. We found out that in networks with sharp in-degree distribution the most determinant property is the CC (or Mot13). The role of CC has been previously highlighted in other types of systems. To name a few, in [61] the degree of local synchrony is suggested to be dictated by CC while the global synchrony is more influenced by PL, and in [62] CC is found superior to PL in affecting the onset of synchronization. The result of [61] was obtained using a pulse-coupled leaky integrate-and-fire model, whereas in [62] the Kuramoto [63] model of oscillating particles was used. High clustering coefficient has also been linked with poorer performance of artificial neural (Hopfield) networks [64], yet experimental studies show that *in vivo* [65] as well as *in vitro* [66] networks possess much greater clustering coefficient than random networks. Similarly to the study at hand, in our earlier work we have found that the amount of network bursts increases with the locality of the network (where also CC is correlated with the locality) [57] in a network of spontaneously active neurons. Our results are backed by [67], where the number of 3-node triangles (comparable to motif 13, see Fig. 1) in the graphs were positively correlated with the mean level of activity in a discrete-state model of neuronal networks. Our results are also in line with [68], where multiple network structures (many of which were similar to ours) consisting of heterogeneous excitatory neuron populations were considered. The authors of [68] found that the local connections encourage (single-cell) bursting in the network, and they propose that the high number of local feed-back loops (which corresponds to a high CC in our terminology) could facilitate the spreading of the bursting activity across the network. Opposite or bilateral results were observed in [53], where the network bursting frequency was either decreasing or increasing with the rewiring probability 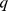 of WS networks (which is anti-correlated with CC), depending on the fraction of the inhibitory population. The differences between their and our results could emerge from the differences in the burst detection procedures. In [53] the burst detection is based on finding the peaks in the smoothened global firing rate, as we applied the burst detection based on maximum inter-spike interval and minimum burst size [56]. We found the latter method more reliable in detecting bursts of variable shape. In addition, it allows the further observation of the burst length and burst size in a straightforward way.

We did not find consistent trends in the importance of other structural measures. The good performance of predictors based on Mot12 (as seen in some simulation settings with binomial in-degree distribution in Fig. 7) is most likely an effect of the high correlation between CC and Mot12. The importance of NB (and hence PL as well) is mostly expressed in the prediction of burst length, but even considering solely burst length it gives the smallest prediction error in fewer cases than CC (in binomial in-degree, Fig. 7) or MEig (in power-law distributed in-degree, Fig. 9C) do. The importance of OD is highlighted in the prediction of spike count and burst count in dense (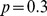) networks with binomial in-degree distribution, but only in HH model, and hence, no conclusions without deeper investigations can be made. Similar observations on the subsidiary effects of the width of the out-degree distribution were reported in [22] and [23].

Another full dimensionality to the aspects of structure-dynamics relationship would be brought about if *modular networks* [69] were studied. In such networks, not only the local connectivity but also the connectivity pattern between the clusters would greatly affect the collective dynamics. This aspect is highly relevant when unraveling the function of a vertebrate brain, and ground-laying studies have already been carried out in the context of, e.g., emergence of sustained activity [67], [70]. Promising attempts were also done in [68], where a biologically inspired modular network model of the mammalian pre-Bötzinger complex was studied by computational means. In their framework, not only the network structure was varied, but also the effect of placing neurons with different intrinsic dynamics (three in total) in different ways was screened. However, we consider that the use of modularly designed networks requires intricate analysis on both intra- and inter-modular connectivities as well as the interplay between them, and leave them to our future studies.

In the generation of the network structure, we fixed the in-degree distribution and allowed the other aspects of the structure to vary. In the framework of [22], all the second-order statistics, which roughly correspond to in-degree deviation, degree correlation, and out-degree deviation, can be controlled in the generation of the graphs. In our framework, the degree correlation and out-degree deviation are affected by the other structural aspects of the network that cannot be ranked by the order of connectivity, such as the proportion of long-range connections in the generation of WS networks. By contrast, networks comparable to FF networks and loopy networks could in principle be generated in a framework similar to [22], but this would require the use of motifs of order higher than 2. In fact, to promote loops of length 6, motifs up to 6th order should be considered, and this might not be computationally feasible. The choice of letting the second order statistics vary could lead to misinterpretations of results if their effect was not screened by other means. Our correlation and prediction framework, by contrast, ensures that the major effect of CC on activity properties in networks with binomial in-degree is not mediated by the other second-order connectivity statistics (or their correlates DC and OD).

It should be noted that we cannot exclude the possibility that there exist measures of structure that perform better than CC and MEig in the prediction of the activity properties. In fact, there may exist a measure that by itself performs better in the prediction task than CC does in networks with sharp in-degree and MEig in networks with wide in-degree distribution. However, the results shown here restrict the properties of such measure – it should be highly correlated with the measures of clustering (CC, Mot13) in networks with binomial in-degree and with measures of degree-degree correlation (MEig, DC) in networks with power-law distributed in-degree. The correlations between graph measures across several network types have been studied in [71], and high correlation coefficient is found between, e.g., the mean of local clustering coefficients (denoted by CC in the present study) and their variance. The high correlation between these measures can also be observed in our network types (correlation coefficient ranges from 0.80 to 0.94, mean 0.90). Indeed, if we replace CC with the standard deviation of the local clustering coefficients, the results in Fig. 7 stay the same. However, if both are included, the mean of local clustering coefficient remains dominating (data not shown).

The activity properties we measured lie in a noisy regime: Multiple runs on an identical connectivity graph results in a great variance in the dynamics (data not explicitly shown, but the trend visible in Fig. 6B). The noisiness of the data is explained by the spontaneous nature of the bursts. As discussed in [53], the dynamical regime that produces the amount of spontaneous activity that is typically seen in neuronal cultures may reside near to the transient from regular to chaotic activity. This transient can be observed in our results in the sparse (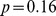) networks with binomial in-degree distribution: These networks lie near the shift from tonic spontaneous spiking to spontaneous bursting activity (the mean burst count is very low in RN, L2, and L4 networks, as seen in Fig. S5). Our results show that both in the regime of numerous and few network bursts the prediction of activity properties using measures of structures *is* feasible. Yet, in some cases the improvements made are not that major, see e.g. the modest difference in prediction errors of the best (black) and the worst (white) predictors of burst count in the densest (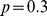) networks in Figs. 6A and 9A. In these networks, the variance of burst count among the networks of same type is considerable, compared to the variability of burst count across the network types (Fig. S5). However, the statistical significance between the prediction errors of different predictors indicates that the fine details of structure still have an effect on the dynamics despite the noisy nature of the bursts.

In the simulations of the excitatory-inhibitory networks, the inhibitory subpopulation was randomly picked once the network structure had been generated. In many brain areas, the connections to and from the inhibitory population obey different connectivity rules than the connections of the excitatory population do. Many *in silico* studies take this diversity into account by applying a specific structure only to the excitatory-excitatory subnetwork, and connecting the excitatory population randomly to the inhibitory population. We conducted our simulations on such networks as well for comparison. The structures of the excitatory subnetworks were first generated by the graph generation algorithms described in the methods section and then randomly coupled to the inhibitory populations. The NETMORPH networks were dismissed from these simulations. The results on the importance of different graph-theoretic measures in predicting activity properties in such networks were qualitatively similar to those reported for EI networks in Figures 7 and 9C (data not shown). Together these results confirm the importance of CC and MEig (of the excitatory subnetwork) also in the presence of inhibition. Due to the different choices of the synaptic weights in excitatory-only and excitatory-inhibitory networks (see Section S1.3 of File S1), which were chosen to restrict the number of bursts on the same range, the difference in the overall network dynamics was not that significant between the two regimes. Nevertheless, several differences exist. In the repetitive simulations of a chosen network type, the spike count varied considerably more in the excitatory-only networks than in excitatory-inhibitory networks (data not shown). Hence, in this sense the inhibitory population brought stability to the total spiking activity of the network. By contrast, the variance in burst length was larger in excitatory-inhibitory networks (data not shown). This could be explained by the many alternatives when and how the inhibitory population can become active during the network burst. Detailed analyses on how the different ways of coupling the inhibitory population to the excitatory population (and the different intrinsic dynamics that the inhibitory neurons may have) affects the network excitability should be carried out in order to draw further conclusions. Moreover, the graph measures should be tuned to consider both the excitatory and inhibitory populations if detailed structure-dynamics relationships were to be uncovered.

Our simulations were conducted in different simulation settings to argue for the generality of our results. The two neuron models applied in this work differ from each other in many aspects: Spiking at the crossing of a threshold membrane potential vs. spiking through the ionic gating variable dynamics; tonic spiking vs. intrinsic bursting; current vs. conductance based synapses; one vs. two excitatory synaptic current components. Although only these two models were used, we believe that similar results would be obtained with any neuron model that represents another combination of these four properties. In both models, the synaptic currents decay exponentially, although in HH model the exact form of the decay is also shaped by the effects of the presynaptic and postsynaptic membrane potentials. A key restriction of our results is that they apply to network bursting that emerges from spontaneous spiking activity in the excitatory neurons. A distinction should be made to synchronization of *rhythmically* active neurons, e.g., the Kuramoto model neurons and the phase response curve model neurons as described in [22]. In such systems, the neurons possess a constant drive toward the “forward” phase, whereas in the models used in this work such a drive is replaced by a white noise current. The effect of CC that is observed in our work could emerge from the deeper need of local integration to attain the network burst. In rhythmically active systems this need may be diminished due to the constant “forward” drive, and hence, only the effect of MEig (or DC) is highlighted in such systems, as discussed in [60] and [22]. Another restriction of our conclusions is that only such models are considered where the network bursts are ceased by the depletion of the excitatory synaptic resources – although in the excitatory-inhibitory networks also the activation of the inhibitory population can contribute to the burst cessation. The first restriction could be relaxed by considering both networks of rhythmically active neurons and networks with spontaneously active neurons, and possibly a continuum between them, while the second restriction cannot be relaxed without applying another mechanism for the cessation of the network bursts. We leave both of these questions to be answered by the future studies.

The focus of this paper is on the bursting properties of networks. No unified theory on the relevance of network bursts has been established, but they have been hypothesized as, e.g., a mechanism of secured information transfer [72], a means for synaptic modification [73], [74], and in the case of power-law distributed burst size, an optimal information transfer and a sign of the network acting in a critical regime [75]. In addition to the nervous system of a maturing (e.g. [76]) and behaving (e.g. [77]) animal, bursts of large populations of neurons are observed in the most primitive neuronal systems such as dissociated neuronal cultures (see for instance [49] or [51]), which emphasizes the fundamentality of the phenomenon. Earlier computational studies enlighten the cellular mechanisms needed for bursting in neuronal networks (see for example [45]), but few pieces of work monitor the effect of network structure on bursting.

Network bursts represent an extreme type of synchronized spiking activity, and understanding which aspects of structure contribute to the emergence of this synchronization is a crucial milestone in structure-dynamics research in neuroscience. The implications of such knowledge are manifold. The future techniques may allow indirect measurements of certain structural properties of the network [12], [27] although the full connectome is unavailable. Knowing which properties are crucial for the network dynamics could help make predictions on the statistics of the activity basing on the measured aspects of the structure. On the other hand, knowledge on structure-dynamics relationship in neuronal networks could be useful in the design of artificial intelligence in the future. The increasing computational power will allow the use of artificial neural networks that are biologically more realistic than the currently existing ones. Given an *in silico* implementation, the designing of the structure in such networks need not be restricted by the physical limitations, such as wiring cost, that exist in their biological counterparts.

## Supporting Information

Figure S1
**In-degree distributions for NM networks with connection probabilities 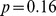 (orange), 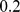 (purple), and 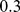 (blue).** The dashed lines show the binomial PDFs with these connection probabilities, and the legend shows the KL-divergence of the NM in-degree distributions from these binomial distributions. The obtained values of 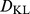 are considerably small – the corresponding values between NM in-degree distributions and the best-fit triangular distribution are 0.22 (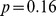), 0.24 (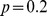), and 0.24 (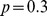), and further, the corresponding 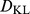 values are 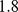, 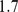 and 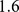 between NM distributions and uniform distribution. The NM in-degree distributions are constructed from a pool of 400 network realizations.(EPS)

Figure S2
**CC in FF networks as a function of parameter 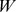, and, for comparison, the respective values of RN, LCN1, LCN2 and NETM networks.** By the increase of 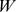 the CC of FF networks approaches that of the extreme FF networks, yet remains lower than that in LCN1, LCN2 or even NM. Different colors represent networks with different (binomial) in-degree distributions. The solid line shows the average over 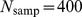 trials, and the shaded area the standard deviation.(EPS)

Figure S3
**Number (mean and std, 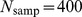) of FF-motifs in FF networks as a function of parameter 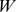, and, for comparison, the respective values of RN, LCN1 and NETM networks.** The in-degree distribution of FF, RN and LCN1 networks is chosen as binomial with the shown average connectivities.(EPS)

Figure S4
**Distribution of eigenvalues 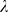 of L2, L3, L4 and L6 networks with parameter 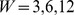 and 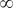.** Different colors represent networks with different binomial in-degree distributions, average connectivities chosen as 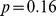 (orange), 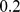 (purple), and 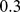 (blue). Each plot shows the combined spectra of 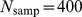 networks. The corresponding spectra for RN, LCN1, LCN2 and NETMORPH networks are plotted for comparison. In the extreme (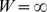) L2, L3, L4 and L6 networks one can observe the division of the eigenvalues to 2, 3, 4 and 6 distinct horns, respectively. The number of horns reflects the frequent occurrence of paths of the corresponding length in the graphs.(EPS)

Figure S5
**Statistics (mean and std) on the activity properties of different network classes.** For the network classes that allow the use of the strength parameter 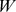 (WS1, WS2, FF, L2, L3, L4 and L6), only the statistics for the extreme networks (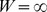) shown.(EPS)

Figure S6
**CC is most the determinant graph property in large networks with binomial in-degree, while MEig is the most relevant in large networks with power-law distributed in-degree.** The upmost panel shows the burst count statistics for the extreme networks, see Fig. S5 for reference. The second and third panels show the prediction errors of burst count in large networks with binomial or power-law distributed in-degree, respectively, and the 2D-plots show the burst count w.r.t. dominant graph measure in mid-dense (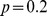) networks. The corresponding data for small networks are shown in Figs. 6 and 9A–B. The lowest panel shows the prediction improvements in large networks, see Figs. 8 and 9D for comparison with small networks. In the repetition of the predictions both the target and teaching data are resampled from the total number of 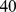 networks.(EPS)

File S1 **Supporting Information.**
(PDF)
