# Supplementary material for: Structure-Dynamics Relationships in Bursting Neuronal Networks Revealed Using a Prediction Framework
Source: PLoS One. 2013 Jul 25;8(7):e69373. doi: 10.1371/journal.pone.0069373 (PMC3723901; doi:10.1371/journal.pone.0069373)
Supplement: File S1 — Supporting Information. (PDF) [file pone.0069373.s007.pdf]

Structure-Dynamics Relationships in Bursting Neuronal Networks Revealed using a Prediction Framework — Supporting information

S1 Supporting information on the methods

S1.1 Network generation algorithms

In this section we show the pseudo-codes for generating the networks with higher occurrence of feed-ferward loops (Algorithm S1) and the networks with higher occurrence of directed loops of length L (Algorithm S2). The MATLAB implementations for the algorithms are given in ModelDB entry 147117. Both algorithms are given the number of nodes N, the in-degree distribution $f_{\mathbf{ID}}$, and the strength parameter W as attributes. The algorithms start with an empty connectivity matrix $M \in \{0,1\}^{N\times N}$ that is updated every time a connection is made, and finally they output M.

Algorithm S1 Scheme for FF networks.

for node index $i \in \{1,\dots,N\}$ **do**
  · Draw number of inputs $n_i \sim f_{\mathbf{ID}}$.
  for input index $j \in \{1,\dots,n_i\}$ **do**
    · Give weights a_k to all nodes $k \neq i$ that do not yet project to i s.t. $a_k = 1 + |\{l|M_{kl} \wedge M_{li}\}|$.
    · Compute the probability to draw node k as $P(k) = \frac{a_k^W}{\sum_k a_k^W}$.
    · Randomly pick k according to the probability mass distribution P and create a connection from k to i.
  end for
end for

In Algorithm S1 all inputs of a node i are set successively before setting the inputs of other nodes. The connectivity graph is updated every time a connection is made, and hence also the probability distribution $P(k)$ of possible inputs changes with every step. These probabilities are given on the basis of the number of disynaptic paths they have to the considered node i. That is, higher priority is given to nodes that project to the input nodes of node i. Conversely, in Algorithm S2 the edges are set in such a way that the node i for which the input is selected is changed in every iteration. By default, the node i is selected as the node that was last chosen as an input to another node. This promotes the creation of chains in the early stage of the iteration, which is crucial for the succesful creation of loops in the later stage. In case the node that was last chosen as input already has all its inputs set, the node to be updated is picked by random on the basis of the number of unset inputs of each node. This is also done in the first iteration of the algorithm.

In addition, the weighting scheme in the picking of inputs in Algorithm S2 is more diverse than that in Algorithm S1. The highest priority ($4+\epsilon_k$ points) is given to such nodes k that, if chosen as an input to i, would create a loop of length L from i to itself without shortcuts. The second highest priority (3) is given to such nodes that would not create a loop of length L, but would not either create a shorter loop from i to itself. The third highest priority ($2+\epsilon'_k$) is given to nodes that would create a loop of length L, but would simultaneously create shorter loops. The lowest priority (1) is given to the rest, i.e., the nodes that, if chosen as input to i, would create a loop shorter than L but would not add loops of length L. Further difference between nodes on the first or third priority level is given by $\epsilon_k = \frac{(M^{L-1})_{ik}}{\left(\frac{N-2}{L-2}\right)^{L-2}}$ and $\epsilon'_k = \frac{(M^{L-1})_{ik}}{(N-1)^{L-2}}$, both of which are proportional to the number of paths of length L that would be formed if the node k was chosen as input. To ensure that these extra terms are subsidiary to the named four

Algorithm S2 Scheme for loopy networks of length L.

for node index $i \in \{1, \ldots, N\}$ **do**
 · Draw number of inputs $n_i \sim f_{\mathbf{ID}}$.
end for
while not all edges set **do**
 if this is the very first edge or if the node that was last selected has already been set all its inputs **then**
  · Give weights b_i to all nodes i such that b_i is the number of inputs of i that have not yet been set.
  · Compute the probability to draw node i as $P(i) = \frac{a_k^W}{\sum_i a_i^W}$.
  · Randomly pick i according to the probability mass distribution P
 else
  · Set i to be the node that was selected as the input node in the last round.
 end if
 For each $l < L$ and each possible input k, calculate $(M^l)_{ik}$, i.e., the number of existing paths of length l from node i to node k. Give weights a_k to all nodes that do not project to i as follows.
 for node index $k \in \{1, \ldots, N\} \setminus \{i\} \setminus \{l | M_{li} = 1\}$ **do**
  if $\forall l = 1, \ldots, L-2 : (M^l)_{ik} = 0$ **then**
   if $(M^{L-1})_{ik} > 0$ **then**
    · Set $a_k = 4 + \frac{(M^{L-1})_{ik}}{\left(\frac{N-2}{L-2}\right)^{L-2}}$
   else
    · Set $a_k = 3$
   end if
  else
   if $(M^{L-1})_{ik} > 0$ **then**
    · Set $a_k = 2 + \frac{(M^{L-1})_{ik}}{(N-1)^{L-2}}$
   else
    · Set $a_k = 1$
   end if
  end if
  · Compute the probability to draw node k as $P(k) = \frac{a_k^W}{\sum_k a_k^W}$.
  · Randomly pick k according to the probability mass distribution P and create a connection from k to i.
 end for
end while

priority levels, we show that $\epsilon_k, \epsilon'_k \leq 1$ as follows.

Proof for ϵ_k:

It can be shown that $(M^{L-1})_{ik} \leq \left(\frac{N-2}{L-2}\right)^{L-2}$. As the choice of k as an input would not create loops shorter than L, the minimum path length from i to k has to be $L-1$. The maximum number of such paths is attained by (if possible) ordering the remaining $N-2$ nodes into $L-2$ layers, each of which contains a maximum of $\lceil (N-2)/(L-2) \rceil$ nodes. In this construction i projects to all nodes in the first layer, the nodes of the first layer nodes project to all nodes in the second layer, and continuing until the last layer, where all nodes project to k. The maximum number of paths of length $L-1$ from i to k is then $(\frac{N-2}{L-2})^{L-2}$.

Proof for ϵ'_k:

We can show by induction that $\forall k : (M^t)_{ik} \leq (N-1)^{t-1}$ in any graph of interest. Since no graph can have more paths than the fully connected graph, that is, a graph M where $M_{ij} = 1 \ \forall j \neq i$ and $M_{ii} = 0$ $\forall i$, it suffices to show the result for that particular graph.

1. The statement is true for $t = 1$, as the number of paths of length 1 from i to any node are either 0 or 1.
2. Suppose the statement is true for $t = t_0 - 1$. Then, the number of paths of length t_0 from i to any k is $\sum_{j=1}^{N} (M^{t_0-1})_{ij} M_{jk} = \sum_{j=1,\ j\neq k}^{N} (M^{t_0-1})_{ij} \leq (N-1)(N-1)^{t_0-1} = (N-1)^{t_0}$. Hence, the statement is true for $t = t_0$.

Thus, $\forall k : \epsilon'_k \leq 1$ in any graph M.

In both algorithms the limit case $W = \infty$ is allowed. In this case, after calculating the weights a_k, the probability mass is divided equally between the nodes that have the exact maximum weight $\max a_k$, and other nodes are given zero probability mass. This can be shown in a simple limit value analysis as follows. Consider the weight for node $k \neq i$.

1. Suppose $\exists j$ such that $a_k < a_j$. Then $\frac{a_k^W}{\sum_{l\neq i} a_l^W} \leq \frac{a_k^W}{a_j^W} = \left(\frac{a_k}{a_j}\right)^W \to 0$. Hence, $\frac{a_k^W}{\sum_{l\neq i} a_l^W} \to 0$.
2. Suppose $a_k = \max\limits_{l\neq i} a_l$. Denote $I = \{l \neq i | a_l = a_k\}$, and denote the size of the set by $n = |I| < N$. If all nodes have the maximum weight a_k, i.e., $n = N-1$, we have $\frac{a_k^W}{\sum_{l\neq i} a_l^W} = \frac{1}{N-1}$, and hence the statement is true. Otherwise, let us consider the remaining nonempty set $J = \{1, \ldots, N\} \setminus \{i\} \setminus I$. Let us choose $j = \arg\max\limits_{l\in J} a_l$. Thus, we have $\frac{a_k^W}{\sum_{l\neq i} a_l^W} = \frac{a_k^W}{\sum_{l\in I} a_l^W + \sum_{l\in J} a_l^W} \geq \frac{a_k^W}{n a_k^W + (N-1-n) a_j^W} = \frac{1}{n + (N-1-n)\left(\frac{a_j}{a_k}\right)^W} \to \frac{1}{n}$. On the other hand we have $\frac{a_k^W}{\sum_{l\neq i} a_l^W} \leq \frac{a_k^W}{n a_k^W} = \frac{1}{n}$. Hence, we have $\frac{a_k^W}{\sum_{l\neq i} a_l^W} \to \frac{1}{n}$.

S1.2 Truncated power-law distribution

In this work, both binomial and power-law distributions are used for the in-degree of the networks. The power-law distribution is truncated as follows:

$$f_{\mathbf{ID}}^{\mathrm{POW}}(n|N,\alpha,p) = \begin{cases} 1 - \sum_{k=n_{\min}}^{N-1} a k^{\alpha}, & n = n_{\min} \\ a n^{\alpha}, & n_{\min} < n < N \\ 0, & \text{otherwise} \end{cases}, \tag{S1}$$

where $n_{\min} \in \mathbb{N}$ and $a \in [0, \infty)$ are chosen such that $p = \frac{1}{N-1} \mathrm{E}_{f_{\mathbf{ID}}^{\mathrm{POW}}}[\mathbf{n}]$, and $n_{\min}$ is minimized with the restriction that $1 - \sum_{k=n_{\min}}^{N-1} a k^{\alpha}$ be non-negative. The slope of the power-law distribution is chosen

$\alpha = -2$ throughout this work, based on the data from functional connectivity graphs [S1]. In the following we show that such a and $n_{\min}$ exist for every $N \in \mathbb{N}$, $\alpha \in \mathbb{R}$, and $p \in [0, 1]$.

Let $N \in \mathbb{N}$ be the number of nodes in the network, $p \in [0, 1]$ the desired connection probability, and $\alpha \in \mathbb{R}$ the slope of the power-law distribution. Let $N > 1$ — otherwise, the connection probability is undefined. Let us show that there exist $a \in [0, \infty)$ and $n_{\min} \in \mathbb{N}$ such that the function

$$g(n|N, \alpha, a, n_{\min}) = \begin{cases} 1 - \sum_{k=n_{\min}+1}^{N-1} ak^{\alpha}, & n = n_{\min} \\ an^{\alpha}, & n_{\min} < n \leq N-1 \\ 0, & \text{otherwise} \end{cases}, \tag{S2}$$

is a probability density function on $\Omega = \{0, 1, \ldots, N-1\}$ and satisfies

$$\mathrm{E}_g[\mathbf{n}] = p(N-1). \tag{S3}$$

First, we find that $g(\cdot|\alpha, a, n_{\min})$ is a well-defined probability density function if and only if $n_{\min} = N - 1$, or $n_{\min} < N - 1$ and $a \in [0, 1/\sum_{k=n_{\min}+1}^{N-1} k^{\alpha}]$. Let us now study the Equation S3.

- If $p = 1$, we find that $n_{\min} = N - 1$ satisfies Equation S3 for any a.
- If $p < 1$, we find that $n_{\min} = N - 1$ cannot satisfy the Equation S3 for any a. Consider now an arbitrary $n_{\min} \in \{0, 1, \ldots, N-2\}$. The expectation value

$$\mathrm{E}_g[\mathbf{n}] = (1 - \sum_{k=n_{\min}+1}^{N-1} ak^{\alpha})n_{\min} + \sum_{k=n_{\min}+1}^{N-1} ak^{\alpha+1} = a\left(\sum_{k=n_{\min}+1}^{N-1} k^{\alpha+1} - n_{\min}\sum_{k=n_{\min}+1}^{N-1} k^{\alpha}\right) + n_{\min}$$

is a monotonically increasing function w.r.t. a. For $a = 0$ we have $\mathrm{E}_g[\mathbf{n}] = n_{\min}$, as for other extreme $a = 1/\sum_{k=n_{\min}+1}^{N-1} k^{\alpha}$ we have

$$\mathrm{E}_g[\mathbf{n}] = \frac{\sum_{k=n_{\min}+1}^{N-1} k^{\alpha+1}}{\sum_{k=n_{\min}+1}^{N-1} k^{\alpha}}. \tag{S4}$$

As in the summation terms the index $k \geq n_{\min} + 1$ and k^{α} is non-negative, we have

$$\sum_{k=n_{\min}+1}^{N-1} k^{\alpha} = \sum_{k=n_{\min}+1}^{N-1} k^{\alpha} \cdot 1 \leq \sum_{k=n_{\min}+1}^{N-1} k^{\alpha}(k - n_{\min}) = \sum_{k=n_{\min}+1}^{N-1} k^{\alpha+1} - n_{\min}\sum_{k=n_{\min}+1}^{N-1} k^{\alpha},$$

which gives us

$$\sum_{k=n_{\min}+1}^{N-1} k^{\alpha+1} \geq (n_{\min} + 1)\sum_{k=n_{\min}+1}^{N-1} k^{\alpha}$$

and further

$$\frac{\sum_{k=n_{\min}+1}^{N-1} k^{\alpha+1}}{\sum_{k=n_{\min}+1}^{N-1} k^{\alpha}} \geq n_{\min} + 1.$$

Hence, the range of expectation values $\mathrm{E}_g[\mathbf{n}]$ covers at least the range $[n_{\min}, n_{\min}+1]$. Hence, there is at least one $n_{\min}$ for which a can be chosen (the choice is unique because of the strict monotonicity) such that Equation S3 is satisfied and $g(\cdot|N, \alpha, a, n_{\min})$ is a probability density function. Since there are a finite number of viable values of $n_{\min}$, we may choose the smallest one and the corresponding a. $\square$

S1.3 The LIF and HH models

The parameters for LIF model are taken from [S2] with the following exceptions. In [S2] the background current I_b has differing values for every neuron, making some neurons in the network intrinsically active pacemakers and others excitable only with positive input. In this work we set all the neurons of a given type (excitatory, inhibitory) to the same mean level by their spontaneous activity. The noise level was chosen such that the neurons express spontaneous spikes with a rate of ≈ 10 spikes/min when no inputs are given. The lack of pacemakers prevents the network from having a prominent heterogeneousity that is not due to the structure of the network. The synaptic weights η are tuned such that a RN with a connection probability $p = 0.2$ produces approximately 10 bursts/min, see the end of this section for exact values. The values of the other synaptic parameters (U, τ_{rec}, τ_{facil}, τ_{I}) are set as the mean value given in [S2] without perturbation. The simulations are carried out in PyNEST with time step $dt = 0.2$ms.

The HH model is taken from the Appendix in [S3] with some retuning for our purposes. Here too we apply random current $\mathbf{I}_b(t)$ to the membrane potential such that the neurons fire spontaneously with a moderate rate (8.1 spikes/min for excitatory neurons and 137 spikes/min for inhibitory neurons; these values fit fairly well to experimental data [S4]). Euler-Maruyama method with time step $dt = 0.0025$ms is used for the integration. The Kdr conductance for excitatory neurons is set $g_{\mathrm{Kdr}} = 6\mathrm{mS/cm}^2$ instead of the $3\mathrm{mS/cm}^2$ stated in [S3] — the value $6\mathrm{mS/cm}^2$ (given in a ModelDB entry corresponding to their article) was found to give the correct shape of action potentials.

The proportions of the synaptic currents are similar to [S3] with the exception that also the inhibitory–inhibitory currents are considered. The dynamics for AMPA currents are modeled according to the Appendix of [S5]. The model for NMDA currents is a combination with both the synaptic depression from [S5] and the dynamics of slow rise-time from [S3]. The values of the parameters, when different, are taken from the Appendix of [S3]. The synaptic depression affects both AMPA and NMDA currents through the amount of glutamate resources $T_{\mathrm{Glu}} \in [0, 1]$. In the following, all the model equations and parameters are listed.

The membrane potential of a LIF neuron obeys

$$C_m \frac{dV_m}{dt} = -\frac{V_m}{R_m} + I_{\mathrm{syn}} + \mathbf{I}_b,$$

with C_m=30pF and R_m=1GΩ. The threshold potential is 15mV, the reset potential is 13.5mV, and the refractory period is 3ms for excitatory neurons and 2ms for inhibitory neurons. The synaptic current I_{syn} to the neuron j is the sum of the currents from the presynaptic cells:

$$I_{\mathrm{syn}}^{j} = \begin{cases} \eta(1.0\mathrm{pA} \cdot \sum_{i=1}^{N_E} M_{ij} y_{ij} - 3.0\mathrm{pA} \cdot \sum_{i=N_E+1}^{N} M_{ij} y_{ij}), \text{if } j \text{ excitatory} \\ \eta(4.0\mathrm{pA} \cdot \sum_{i=1}^{N_E} M_{ij} y_{ij} - 4.0\mathrm{pA} \cdot \sum_{i=N_E+1}^{N} M_{ij} y_{ij}), \text{if } j \text{ inhibitory} \end{cases},$$

where the neurons are assumed to be ordered such that the excitatory population consists of neurons with indices $1, \ldots, N_E$ and the inhibitory population of neurons $N_E+1, \ldots, N_E+N_I$. Thus, the synaptic currents are functions of the dynamic synaptic variables y_{ij}, each of which is determined by the following

set of equations:

$$\begin{aligned}\frac{du}{dt} &= -\frac{u}{\tau_{\text{facil}}} + U(1-u)\delta_{t_{\text{sp}}}(t)\\ \frac{dx}{dt} &= \frac{z}{\tau_{\text{rec}}} - ux\delta_{t_{\text{sp}}}(t)\\ \frac{dy}{dt} &= -\frac{y}{\tau_I} + ux\delta_{t_{\text{sp}}}(t)\\ \frac{dz}{dt} &= \frac{y}{\tau_I} - \frac{z}{\tau_{\text{rec}}},\end{aligned} \tag{S5}$$

where t_{sp} is the time instant of a presynaptic spike. The parameters are as follows: $U(E \to E) = U(I \to E) = 0.5$, $U(E \to I) = U(I \to I) = 0.04$, $\tau_{\text{rec}}(E \to E) = \tau_{\text{rec}}(I \to E) = 800\text{ms}$, $\tau_{\text{rec}}(E \to I) = \tau_{\text{rec}}(I \to I) = 100\text{ms}$, $\tau_I(E \to E) = \tau_I(I \to E) = \tau_I(E \to I) = \tau_I(I \to I) = 3\text{ms}$, $\tau_{\text{facil}}(E \to E) = \tau_{\text{facil}}(I \to E) = 0\text{ms}$, $\tau_{\text{facil}}(E \to I) = \tau_{\text{facil}}(I \to I) = 1000\text{ms}$. The background current $\mathbf{I}_b$, chosen independently for each neuron, is a stepwise constant random (Gaussian) current with mean 12pA and standard deviation 7.3pA. The value of the background current is re-initialized every 1ms.

The time course of an excitatory HH neuron is determined by

$$C_m \frac{dV_m}{dt} = -I_{\text{Na}} - I_{\text{NaP}} - I_{\text{Kdr}} - I_{\text{K-slow}} - I_{\text{L}} - I_{\text{AMPA}}^{\text{E}\to\text{E}} - I_{\text{NMDA}}^{\text{E}\to\text{E}} - I_{\text{GABA}} + \mathbf{I}_b,$$

where $C_m = 1\mu\text{F/cm}^2$. The sodium currents I_{Na} obey the following equations:

$$\begin{aligned}I_{\text{Na}}(V_m, h) &= g_{\text{Na}} m_\infty^3(V_m) h (V_m - V_{\text{Na}})\\ \frac{dh}{dt} &= (h_\infty(V_m) - h)/\tau_h(V_m)\\ m_\infty(V) &= (1 + \exp(-(V - \theta_m)/\sigma_m))^{-1}\\ h_\infty(V) &= (1 + \exp(-(V - \theta_h)/\sigma_h))^{-1}\\ \tau_h(V) &= 0.1 + 0.75 \cdot (1 + \exp(-(V - \theta_{th})/\sigma_{th}))^{-1}\\ I_{\text{NaP}}(V_m) &= g_{\text{NaP}} p_\infty(V_m)(V_m - V_{\text{Na}})\\ p_\infty(V) &= (1 + \exp(-(V - \theta_p)/\sigma_p))^{-1},\end{aligned}$$

where $g_{\text{Na}} = 35\text{mS/cm}^2$, $V_{\text{Na}} = 55\text{mV}$, $\theta_m = -30\text{mV}$, $\sigma_m = 9.5\text{mV}$, $\theta_h = -45\text{mV}$, $\sigma_h = -7\text{mV}$, $\theta_{th} = -40.5\text{mV}$, $\sigma_{th} = -6\text{mV}$, $g_{\text{NaP}} = 0.2\text{mS/cm}^2$, $\theta_p = -47\text{mV}$, and $\sigma_p = 3\text{mV}$. The potassium currents are described by the following equations:

$$\begin{aligned}I_{\text{Kdr}}(V_m, n) &= g_{\text{Kdr}} n^4 (V_m - V_{\text{K}})\\ \frac{dn}{dt} &= (n_\infty(V_m) - n)/\tau_n(V_m)\\ n_\infty(V) &= (1 + \exp(-(V - \theta_n)/\sigma_n))^{-1}\\ \tau_n(V) &= 0.1 + 0.5 \cdot (1 + \exp(-(V - \theta_{tn})/\sigma_{tn}))^{-1}\\ I_{\text{K-slow}}(V_m, z) &= g_{\text{K-slow}} z (V_m - V_{\text{K}})\\ \frac{dz}{dt} &= (z_\infty(V_m) - z)/\tau_z\\ z_\infty(V) &= (1 + \exp(-(V - \theta_z)/\sigma_z))^{-1},\end{aligned}$$

where $g_{\rm Kdr}=6{\rm mS/cm}^2$, $V_{\rm K}=-90{\rm mV}$, $\theta_n=-33{\rm mV}$, $\sigma_n=10{\rm mV}$, $\theta_{tn}=-27{\rm mV}$, $\sigma_{tn}=-15{\rm mV}$, $g_{\rm K-slow}=1.8{\rm mS/cm}^2$, $\theta_z=-39{\rm mV}$, $\sigma_z=5{\rm mV}$, and $\tau_z=75{\rm ms}$. The leak current obeys

$$I_{\rm L}(V_m)=g_{\rm L}(V_m-V_{\rm L}),$$

where $g_{\rm L}=0.05{\rm mS/cm}^2$ and $V_{\rm L}=-70{\rm mV}$.

Inhibitory neurons in networks of HH neurons are described by the Wang-Buzsaki model as follows:

$$C_m\frac{dV_m}{dt}=-I^{\rm I}_{\rm Na}-I^{\rm I}_{\rm Kdr}-I^{\rm I}_{\rm L}-I^{\rm E\to I}_{\rm AMPA}-I^{\rm E\to I}_{\rm NMDA}-I_{\rm GABA}+\mathbf{I}_b.$$

The currents are described as follows:

$$\begin{aligned}
I^{\rm I}_{\rm Na}(V_m,h)&=g^{\rm I}_{\rm Na}(m^{\rm I}_\infty(V_m))^3h(V_m-V^{\rm I}_{\rm Na})\\
\frac{dh}{dt}&=\alpha_h(V_m)(1-h)-\beta_h(V_m)h\\
m^{\rm I}_\infty(V)&=\alpha_m(V)/(\alpha_m(V)+\beta_m(V))\\
\alpha_h(V)&=0.35\exp(-(V+58)/20)\\
\beta_h(V)&=5/(1+\exp(-(V+28)/10))\\
\alpha_m(V)&=0.5(V+35)/(1-\exp(-(V+35)/10))\\
\beta_m(V)&=20\exp(-(V+60)/18)\\
I^{\rm I}_{\rm Kdr}(V_m,n)&=g^{\rm I}_{\rm Kdr}n^4(V_m-V^{\rm I}_{\rm K})\\
\frac{dn}{dt}&=\alpha_n(V_m)(1-n)-\beta_n(V_m)n\\
\alpha_n(V)&=0.05(V+34)/(1-\exp(-(V+34)/10))\\
\beta_n(V)&=0.625\exp(-(V+44)/80)\\
I^{\rm I}_{\rm L}(V_m)&=g^{\rm I}_{\rm L}(V_m-V^{\rm I}_{\rm L}),
\end{aligned}$$

with $g^{\rm I}_{\rm Na}=35{\rm mS/cm}^2$, $V^{\rm I}_{\rm Na}=55{\rm mV}$, $g^{\rm I}_{\rm Kdr}=9{\rm mS/cm}^2$, $V^{\rm I}_{\rm K}=-90{\rm mV}$, $g^{\rm I}_{\rm L}=0.1{\rm mS/cm}^2$, and $V^{\rm I}_{\rm L}=-65{\rm mV}$.

The synaptic currents in the HH model are the AMPA and NMDA currents elicited by excitatory neurons and the GABA currents elicited by the inhibitory neurons. The AMPA and NMDA currents express synaptic depression [S5]. For an excitatory neuron ($j\le N_e$), they can be described as follows:

$$\begin{aligned}
I^{\rm E\to E}_{{\rm AMPA},j}(V_m,\{s_{\rm AMPA}\})&=g^{\rm E\to E}_{\rm AMPA}(V_m-V_{\rm Glu})\eta\sum_{i=1}^{N_E}M_{ij}s_{{\rm AMPA},i}\\
\frac{ds_{{\rm AMPA},i}}{dt}&=k_{fP}T_{{\rm Glu},i}s_\infty(V_i)(1-s_{{\rm AMPA},i})-s_{{\rm AMPA},i}/\tau_{\rm AMPA}\\
\frac{dT_{{\rm Glu},i}}{dt}&=-k_ts_\infty(V_i)T_{{\rm Glu},i}+k_v(1-T_{{\rm Glu},i})\\
s_\infty(V)&=(1+\exp(-(V-\theta_s)/\sigma_s))^{-1},\\
I^{\rm E\to E}_{{\rm NMDA},j}(V_m,\{s_{\rm NMDA}\})&=g^{\rm E\to E}_{\rm NMDA}f_{\rm NMDA}(V_m)(V_m-V_{\rm Glu})\eta\sum_{i=1}^{N_E}M_{ij}s_{{\rm NMDA},i}\\
\frac{{\rm d}x_{{\rm NMDA},i}}{{\rm d}t}&=k_{xN}s_\infty(V_i)(1-x_{{\rm NMDA},i})-(1-s_\infty(V_i))x_{{\rm NMDA},i}/\tilde\tau_{\rm NMDA}\\
\frac{{\rm d}s_{{\rm NMDA},i}}{{\rm d}t}&=k_{fN}T_{{\rm Glu},i}x_{{\rm NMDA},i}(1-s_{{\rm NMDA},i})-s_{{\rm NMDA},i}/\tau_{\rm NMDA}\\
f_{\rm NMDA}(V)&=(1+\exp(-(V-\theta_{\rm NMDA})/\sigma_{\rm NMDA}))^{-1},
\end{aligned}$$

where $\theta_s = -20$mV, $\sigma_s = 2$mV, $k_{fP} = 1\text{ms}^{-1}$, $\tau_{\text{AMPA}} = 5$ms, $g^{\text{E}\to\text{E}}_{\text{AMPA}} = 0.08\text{mS/cm}^2$, $V_{\text{Glu}} = 0$mV, $k_t = 1\text{ms}^{-1}$, $k_v = 0.001\text{ms}^{-1}$, $k_{xN} = 1\text{ms}^{-1}$, $\tilde{\tau}_{\text{NMDA}} = 14.3$ms, $k_{fN} = 1\text{ms}^{-1}$, $\tau_{\text{NMDA}} = 100$ms, $g^{\text{E}\to\text{E}}_{\text{NMDA}} = 0.07\text{mS/cm}^2$, and $\sigma_{\text{NMDA}} = 10$mV. The value of θ_{NMDA} is dependent on the magnesium concentration as follows: $\theta_{\text{NMDA}} = 10.5\text{mV} \cdot \ln([\text{Mg}^{2+}]_o/38.3\text{mM})$. Here, the magnesium concentration of $[\text{Mg}^{2+}]_o = 0.7$mM is used, which is a typical value in cortical cultures.

The AMPA and NMDA currents to inhibitory neurons ($j > N_E$) differ from the corresponding currents to excitatory neurons only through the synaptic conductances:

$$I^{\text{E}\to\text{I}}_{\text{AMPA},j}(V_m, \{s_{\text{AMPA}}\}) = g^{\text{E}\to\text{I}}_{\text{AMPA}}(V_m - V_{\text{Glu}})\eta \sum_{i=1}^{N_E} M_{ij} s_{\text{AMPA},i}$$
$$I^{\text{E}\to\text{I}}_{\text{NMDA},j}(V_m, \{s_{\text{NMDA}}\}) = g^{\text{E}\to\text{I}}_{\text{NMDA}} f_{\text{NMDA}}(V_m)(V_m - V_{\text{Glu}})\eta \sum_{i=1}^{N_E} M_{ij} s_{\text{NMDA},i} \quad \text{(S6)}$$

where $g^{\text{E}\to\text{I}}_{\text{AMPA}} = 0.2\text{mS/cm}^2$ and $g^{\text{E}\to\text{I}}_{\text{NMDA}} = 0.05\text{mS/cm}^2$. The GABA currents to both excitatory and inhibitory neurons are described as follows:

$$I_{\text{GABA},j}(V_m, \{s_{\text{GABA}}\}) = g_{\text{GABA}}(V_m - V_{\text{GABA}})\eta \sum_{i=N_E+1}^{N} M_{ij} s_{\text{GABA},i}$$
$$\frac{ds_{\text{GABA},i}}{dt} = k_{fA} s_\infty(V_i)(1 - s_{\text{GABA},i}) - s_{\text{GABA},i}/\tau_{\text{GABA}},$$

where $k_{fA} = 1\text{ms}^{-1}$, $\tau_{\text{GABA}} = 10$ms, $g_{\text{GABA}} = 0.05\text{mS/cm}^2$, and $V_{\text{GABA}} = -70$mV. The background current is a zero-mean Brownian white noise term, described as $\mathbf{I}_b(t) = 0.9\mu\text{A/cm}^2 \cdot \mathbf{W}_t$, where $\mathbf{W}_t$ is the independent Wiener process (the "time derivative" of dimensionless Brownian motion).

The initial state of the system is chosen such that all neurons are at or near rest. In LIF model this is done by setting all membrane potentials to the reset value, and in HH model the membrane potential and gating variable values are given by steady states of unconnected, noiseless neurons. In the beginning all synaptic resources are set to the maximum value ($x = 1$ and $y = z = 0$ in LIF model, and $T_{\text{Glu}} = 1$ in HH model).

The values of the synaptic weights η used in this work are given below:

	HH, $N = 100$	LIF, $N = 100$	LIF, $N = 900$
E	0.144	14.52	2.81
EI	0.177	17.21	

S2 Supporting results on network structure

S2.1 NETMORPH networks have roughly binomial in-degree distribution

Fig. S1 shows that the in-degree distribution in 2-dimensional NETMORPH networks with continuous boundaries is fairly well approximated by binomial distribution. This is opposite to 2-dimensional NETMORPH networks without the boundary continuity that are characterised by broader in-degree distribution, as we have shown in [S6]. Hence, we consider NM networks comparable to networks with binomial in-degree.

S2.2 FF networks have high number of feed-forward loops but relatively low clustering coefficient

Fig. S2 shows the clustering coefficient of FF networks as a function of parameter W, together with the RN, LCN1, LCN2, and NM networks. One can observe the rising of the CC in FF networks with the parameter W, which yet remains lower than the CC in locally connected networks LCN1 and LCN2. Fig. S3 in turn shows the number of FF-motifs in FF networks with varying parameter W, and for comparison the corresponding number in RN, LCN1, and NM networks. The number of FF-motifs is calculated as the number of such ordered triples, whose edges form the functional form of motif 5 (see Fig. 1), i.e. $|\{(i,j,k) \in \{1,...,N\}^3 | M_{ij} \wedge M_{jk} \wedge M_{ik}\}|$. The amount of FF-motifs in FF networks is increased with the increase of parameter W. The abundance of FF-motifs in LCN1 networks is explained by the frequent occurrence of motif 13, each of which in itself contains six permutations of the FF-motif. The extreme FF networks acquire a comparable number of FF-motifs, yet they preserve the relatively low degree of clustering, as seen in Fig. S2.

S2.3 Loopy networks express loops in their eigenvalue spectra

One way to illustrate the occurrences of loops in a graph is to plot the eigenvalue spectrum of the connectivity matrix into the complex plane. Consider as an example a perfect ring graph M, consisting of N nodes, where each node has exactly one input and one output, and where the nodes form a traversable ring. The Nth power of the underlying connectivity matrix is an identity matrix, in which all eigenvalues are 1. By basic linear algebra, these eigenvalues must be the Nth powers of the eigenvalues of M, and hence the eigenvalues of M are evenly distributed on the unit circle in $\mathbb{C}$. Fig. S4 shows the eigenvalue spectra of L2, L3, L4 and L6 networks with different parameters W, and for comparison, the eigenvalue spectra of RN, LCN and NM networks. In the extreme cases $W = \infty$ the division of the eigenvalues to 2, 3, 4 or 6 horns is evident. This is fairly non-trivial in the case of denser L6 networks: Since the connection probability is as high as 0.2 or 0.3, it would be reasonable that the abundance of connections break the loopy structure of the network. This abundance does bring up malformations in the star-shaped spectra in the case of $p = 0.3$, but not notably in the case of $p = 0.2$.

S2.4 The relation of mean node-betweenness and mean shortest path length

The mean value of node-betweenness correlates highly with the mean path length. In this section we will analytically derive the connection of these two quantities. The mean betweenness value is calculated as

$$\mathrm{NB} = \frac{1}{N}\sum_{i=1}^{N} \mathrm{NB}_i = \frac{1}{N}\sum_{i=1}^{N} \sum_{\substack{j=1 \\ j \neq i}}^{N} \sum_{\substack{k=1 \\ i \neq k \neq j \\ \mathrm{PL}_{jk} < \infty}}^{N} \frac{s_{jk}^{(i)}}{s_{jk}^{(\mathrm{tot})}}. \tag{S7}$$

The term $s_{jk}^{(i)}$ is the number of such shortest paths from j to k where the node i lies on, and $s_{jk}^{(\mathrm{tot})}$ represents the total number of shortest paths from j to k. The nodes k where no path from j to k exists are excluded from the summation, and hence the quantity is well-defined for any graph.

Let us denote the length of shortest path from j to k by L_{jk}. A necessary and sufficient condition for i lying on the shortest path from j to k is that there be a path of length $l \in \{1, \ldots, L_{jk} - 1\}$ from j to i and a path of length $L_{jk} - l$ from i to k. The total number of such paths can be counted as the product of the number of paths of length l from j to i and the number of paths of length $L_{jk} - l$ from i to k. This can be expressed using the elements of the lth and $(L_{jk} - l)$th exponent of the connectivity matrix

as $(M^l)_{ji} \cdot (M^{L_{jk}-l})_{ik}$. Hence, the total number of shortest paths from j through i to k is calculated by summing this over all possible lengths l as

$$s_{jk}^{(i)} = \sum_{l=1}^{L_{jk}-1} (M^l)_{ji} \cdot (M^{L_{jk}-l})_{ik},$$

while the total number of shortest paths is simply

$$s_{jk}^{(\mathrm{tot})} = (M^{L_{jk}})_{jk}.$$

These can be substitued into Eq. S7, and changing the order of summation gives us

$$\mathrm{NB} = \frac{1}{N} \sum_{j=1}^{N} \sum_{\substack{k=1 \\ k \neq j \\ \mathrm{PL}_{jk} < \infty}}^{N} \sum_{l=1}^{L_{jk}-1} \sum_{\substack{i=1 \\ j \neq i \neq k}}^{N} \frac{(M^l)_{ji} \cdot (M^{L_{jk}-l})_{ik}}{(M^{L_{jk}})_{jk}}.$$

Ordered this way, we notice that the summation over running variable i is actually nothing more than the matrix multiplication of M^l and $M^{L_{jk}-l}$. The elements corresponding to $i = j$ and $i = k$ can be excluded, as they cannot contribute to the summed value. If they did, i.e., if $(M^l)_{jj} \cdot (M^{L_{jk}-l})_{jk}$ or $(M^l)_{jk} \cdot (M^{L_{jk}-l})_{kk}$ were greater than zero, then there would exist a path of length $l < L_{jk}$ or $L_{jk} - l < L_{jk}$ from j to k, contradicting with our definition of L_{jk}. Thereby, we are left with the formula for average node-betweenness

$$\mathrm{NB} = \frac{1}{N} \sum_{j=1}^{N} \sum_{\substack{k=1 \\ k \neq j \\ \mathrm{PL}_{jk} < \infty}}^{N} \sum_{l=1}^{L_{jk}-1} \frac{(M^{L_{jk}})_{jk}}{(M^{L_{jk}})_{jk}} = \frac{1}{N} \sum_{j=1}^{N} \sum_{\substack{k=1 \\ k \neq j \\ \mathrm{PL}_{jk} < \infty}}^{N} (L_{jk} - 1).$$

Hence, the mean node-betweenness is proportional to the mean (non-harmonic, infinite path lengths excluded) path length substracted by the overall proportion of pairs connected by a path of edges. The non-harmonic mean path length in turn correlates with the harmonic mean path length: They are both generalized power means of the same data with exponents 1 and -1, respectively.

S3 Supporting data on network simulations

Fig. S5 shows an overview of the activity properties obtained for different extreme ($W = \infty$) networks. The properties shown are the spike count (SC), the burst count (BC), the average burst length (BL), and the average number of spikes in a burst, i.e., the burst size (BS). Each bar shows the mean and standard deviation of the named activity property in 150 network simulations. The results of purely excitatory (E) networks with medium connectivity ($p = 0.2$), modeled by HH, is shown for all activity properties and both in-degree distributions. The statistics of burst count in the excitatory-inhibitory (EI) networks, networks with different connection probabilities, and LIF networks are shown for reference.

The difference in network activity between the network classes (RN, LCN1, LCN2, FF, L2, L3, L4, L6 and NM) is evident. The main trend in networks with binomially distributed in-degree is that the LCN1, LCN2 and NM networks produce the most networks bursts and also the longest bursts. However, in networks with power-law distributed in-degree some of the loopy networks express higher burst count than LCN1s. In addition, the variance of burst count is less negligible than in their counterparts with

binomial in-degree, suggesting that considering a network class as a unity may not be feasible. This serves as an extra motivation for exploring graph theoretic properties of the networks and their contribution to the dynamics.

Fig. S6 shows the results of the prediction framework for larger (N=900) networks. The different panels correspond to the results shown in Figs. S5, 6, 8, and 9. The figure justifies that our conclusions hold for the bigger networks as well.

References

S1. Eguiluz V, Chialvo D, Cecchi G, Baliki M, Apkarian A (2005) Scale-free brain functional networks. Physical Review Letters 94: 18102.

S2. Tsodyks M, Uziel A, Markram H (2000) Synchrony generation in recurrent networks with frequency-dependent synapses. Journal of Neuroscience 20: 1–5.

S3. Golomb D, Shedmi A, Curtu R, Ermentrout G (2006) Persistent synchronized bursting activity in cortical tissues with low magnesium concentration: A modeling study. Journal of Neurophysiology 95: 1049–1067.

S4. Klostermann O, Wahle P (1999) Patterns of spontaneous activity and morphology of interneuron types in organotypic cortex and thalamus-cortex cultures. Journal of Neuroscience 92: 1243–1259.

S5. Golomb D, Amitai Y (1997) Propagating neuronal discharges in neocortical slices: Computational and experimental study. Journal of Neurophysiology 78: 1199–1211.

S6. Mäki-Marttunen T, Havela R, Aćimović J, Teppola H, Ruohonen K, et al. (2010) Modeling growth in neuronal cell cultures: Network properties in different phases of growth studied using two growth simulators. In: Proceedings of the 7th International Workshop on Computational Systems Biology (WCSB 2010).
